# Vascular flora of Kenya, based on the Flora of Tropical East Africa

**DOI:** 10.3897/phytokeys.90.20531

**Published:** 2017-11-16

**Authors:** Yadong Zhou, Bing Liu, Yuvenlis Mbuni, Xue Yan, Geoffrey Mwachala, Gugangwan Hu, Qingfeng Wang

**Affiliations:** 1 Wuhan Botanical Garden, Chinese Academy of Sciences, Wuhan 430074, Hubei, China; 2 Sino-Africa Joint Research Center, Chinese Academy of Sciences, Wuhan 430074, Hubei, China; 3 State Key Laboratory of Systematic and Evolutionary Botany, Institute of Botany, Chinese Academy of Sciences, Beijing 100093, China; 4 East African Herbarium, National Museums of Kenya, P. O. Box 45166 00100 Nairobi, Kenya

**Keywords:** East Africa, Kenya, FTEA, vascular plants, molecular systematics, diversity

## Abstract

Kenya, an African country with major higher plant diversity, has a corresponding diversity of plant associations, because of the wide geographic distribution, diverse climatic conditions and soil types. In this article, all vascular plants of Kenya were counted based on the completed "Flora of Tropical East Africa (FTEA)", and all families and genera were revised using recent molecular systematics research, forming a "Synoptic List of Families and Genera of Kenyan Vascular Plants (SLFGKVP)". In total, there are 225 families, 1538 genera and 6293 indigenous species and and 62 families, 302 genera and 588 exotic species in Kenya. The Fabaceae with 98 genera and 576 Species is the largest family. Two of the seven plant distribution regions of Kenya, K4 and K7 are the most species-richest areas with regard to both total and endemic species, with 3375 and 3191 total species and 174 and 185 endemic species in K4 and K7 respectively. While, K3 and K5 have the highest density of both total and endemic species. K1 has the lowest density of total species, and K2 has the lowest density of endemic species.

## Introduction

**Figure 1.**
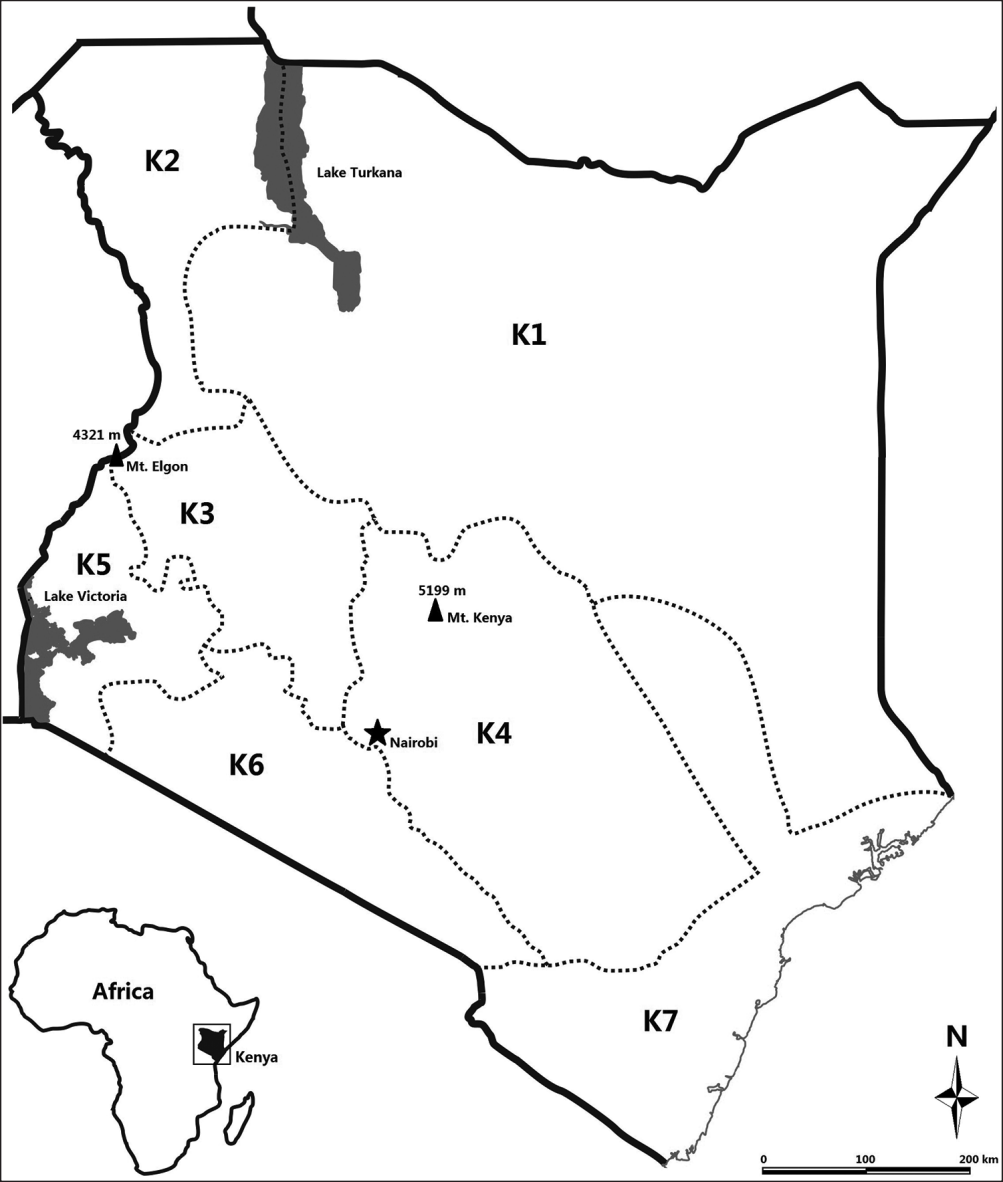
Kenya and its location. **K1–K7** indicates plant distribution regions in "*Flora of Tropical East Africa*" (FTEA editors 1952–2012).

The Republic of Kenya (Fig. 1) lies in the equatorial zone of eastern part of Africa continent, between latitudes 4°N and 4°S and between longitudes 34°E and 42°E. It is bordered by Tanzania to the south, Uganda to the west, Ethiopia to the north, Sudan to the north-west, Somalia to the east, and the Indian Ocean to the south-east (Orodho 2006). Kenya covers a total area of 582,646 km^2^, including 569,253 km^2^ land area and 13,393 km^2^ water area. The equator runs through the central part of Kenya, intersecting the Great Rift Valley, forming the famous "East Africa Cross". Mt. Kenya is the largest ancient extinct volcano in Great Rift Valley areas. It is the highest mountain of Kenya and the second highest in Africa with a height of 5,199 m a.s.l. (the first one being Mt. Kilimanjaro, 5,892 m a.s.l.) (Ojany and Ogendo 1973, Speck 1982, Sombroek et al. 1982, M,U 2009).

According to the early administrative division of Kenya, seven plant distribution regions (K1–K7) had been divided by "Flora of Tropical East Africa" (hereafter FTEA) (FTEA editors 1952–2012) (Fig. 1). K1 represents the "Northern Frontier Province" which is located in the northern part of Kenya. K2 represents the "Turkana Province" which is located in the northwest part of Kenya. K3 represents the "Rift Valley Province" which is located in western part of Kenya. K4 represents the "Central Province" which is located in central part of Kenya. K5 represents the "Nyanza Province" which is located in southwest part of Kenya. K6 represents the “Masai Province” which is located in southern part of Kenya. K7 represents the "Coast Province" which is located along the coastal area of Kenya (Polhill 1988).

The soils of Kenya are diverse ranging from the coral types on the coastal areas to the alluvial, swampy, and black cotton soils along river valleys and plains, to abundant volcanic soils on the high mountain regions (Orodho 2006). Climatically Kenya is considered part of the tropical monsoon area belonging to the tropical savanna climate region, and the annual maximum temperature is from 22 to 26 °C, and annual minimum is from 10 to 14 °C (Gao 2004). Rainfall patterns varies with different seasons around the year, that is, most parts experience high rainfall from March to June and October to December, and the rest are dry spells. Annual rainfall from the southwest to the northeast decreasing from 2000 mm to 250 mm (Gao 2004, Orodho 2006).

Topography, climatic conditions, soil types and human activity, all have a great impact on the vegetation types in Kenya. Three main types and several sub-types of Kenyan vegetation had been recognized by Edwards (1940): i) Forest types, subdividing into mountain forest, thorn forest, and mangrove forest; ii) Grasslands (including parkland or savannah grasslands), subdividing into mountain grassland, high moisture savannah, *Acacia*-tall grass savannah and open grassland (tall grass); iii) Semi-arid grasslands, subdividing into *Acacia*-desert grass savannah and open grassland (desert grass), and desert shrub-desert grass. In recent years, researchers from University of Copenhagen and The World Agroforestry Centre constructed the Potential Natural Vegetation of eight countries from eastern and southern Africa (VECEA team 2015).

The diverse vegetation types of Kenya include numerous indigenous plant species, many of which are endemic, such as *Dendrosenecio keniensis* (Asteraceae) in K4 (Mt. Kenya), *Impatiens fischeri* (Balsaminaceae) in K3 and K4, *Habenaria keniensis* (Orchidaceae) in K3, K4 and K5, and the recently published species *Sedum keniense* (Crassulaceae) in K4 (Mt. Kenya) (Zhou et al. 2016a). Over the years, botanists from all over the world have studied Kenyan plants and accumulated a series of monographs. In 1961, Dale I.R. and Greenway P.J. published "Kenya Trees and Shrubs", which recorded about 1000 trees and shrubs of Kenya (Dale and Greenway 1961), and Beentje H.J. republished this book in 1994 reviewing some species and adding lianas (Beentje 1994). In 1974, Agnew A.D.Q. published "Upland Kenya Wild Flowers" and republished it in 1994, recording over 3000 herbs and ferns of Kenyan upland areas; the third edition of which was published in 2013, with the addition of grasses and sedges (Agnew 1974, 2013, Agnew and Agnew 1994). Other books on Kenya plants include, "The wild flowers of Kenya" (Blundell, 1982), "An illustrated manual of Kenya Grasses"(Ibrahim and Kabuye 1987), "The beautiful plants of Kenya" (Karmali 1988), "Orchids of Kenya" (Stewart 2003), "Useful trees and shrubs for Kenya" (Maundu and Tengnas 2005), "Common plants of Kenya" (Wang et al. 2016), and some other books with a broader coverage of whole east Africa, such as "Collins guide to the wild flowers of East Africa" (Blundell 1987), "Field Guide to Acacias of East Africa" (Dharani 2006), "Medicinal Plants of East Africa, 3^rd^ Edition" (Kokwaro 2009), "Medicinal Plants of East Africa-An Illustrated Guide" (Dharani and Yenesew 2010), "Field Guide to Common Trees and Shrubs of East Africa, 2^nd^ Edition" (Dharani 2011). "Flora of Tropical Africa" was the first flora record of Kenya plants, which included 13000 plants of tropical Africa and divided into 10 volumes (Daniel et al. 1868–1933). "Flora of Tropical East Africa (FTEA)" covers plants from Uganda, Kenya and Tanzania, which has been edited several times since 1952 and completed in 2012. To date, FTEA records c.125000 plant species from the three countries. Shortcomings of FTEA on Kenya plants are that: i) taxa are still under-represented; ii) New families, genera and taxa have not been reviewed and recorded; iii) Families have not been arranged using the recent available botanical systems.

Plant systematics has been one of the hottest research areas in botanical studies (Singh 2010, Simpson 2010). In the early years, some plants systems were published based on morphological characteristics, such as Ching"s system on ferns (Ching 1978a, b), Cheng"s system about gymnosperms (Cheng and Fu 1978), and Takhtanjan"s system, Cronquist"s system and Engler"s system about angiosperms (Yang et al. 2004, Liu et al. 2015). Lycophytes had been separated from ferns according to recent molecular systematics evidences, and was positioned as the sister group to Euphyllophytes (including Spermatophytes and Monilophytes) (Pryer et al. 2004, Liu et al. 2009). Smith et al. (2006) proposed a new classification system on Monilophytes, dividing ferns into 4 classes, 10 orders and 37 families, and later some new families were established and new classification system was proposed (Christenhusz et al. 2011b, Liu et al. 2013, Zhang and Zhang 2015). Recently, the Pteridophyte Phylogeny Group proposed a community-derived classification on extant lycophytes and ferns, including 2 classes, 14 orders and 51 families (PPG I 2016). Based on branch taxonomy and molecular systematics results, the Angiosperm Phylogeny Group (APG) proposed a new classification, refined over four publications (APG 1998, APG II 2003, APG III 2009, APG IV 2016). Compared with the APG III system, the APG IV system proposed two additional informal major clades, namely superrosids and superasterids, and recognized 5 new orders, namely Boraginales, Dilleniales, Icacinales, Metteniusales and Vahliales, resulting in a total of 64 orders and 416 families of Angiosperms (APG IV 2016).

This article summarizes families and genera of Kenya plants based on FTEA, and combined with other recent molecular systematic research. Our goal was to answer the following questions: i) What is the total number of plant species found in Kenya? ii) Which families and genera are the largest? iii) Which part in Kenya contains the highest plant species richness, and iv) What measures can be carried out so as to protect and conserve the Kenyan plants?

## Data and methods

All information is sourced from the FTEA, since it has recorded over 12500 vascular plants of Uganda (U1–U4), Kenya (K1–K7) and Tanzania (T1–T8), including some planted and naturalized species. Each family has been published in one volume, although some big families with 2–4 volumes also have been done, such as Leguminosae (4 volumes), Poaceae (3 volumes), Rubiaceae (3 volumes) and Compositae (3 volumes). Except for some exotic plants, FTEA has described each species, the distribution regions, and characteristic habitat. and recorded the voucher specimens.

Here, we conducted a census of all Kenyan species recorded by FTEA, gathering distribution information on endemic plants, exotic plants and naturalized species. We then reviewed the families and genera of all the species based on recent systematic research, creating a "Synoptic List of Families and Genera of Kenyan Vascular Plants (SLFGKVP)" (see Supplementary material 1). Families of Lycophytes and monilophytes are revised by PPG I system (PPG I 2016), families of gymnosperms are revised the system advocated by Christenhusz et al. (2011a), and families of angiosperms are revised by APG IV system (APG IV 2016). We analyzed the largest families and genera, as well as the number of endemic and exotic species of Kenya plants. We also compared total and endemic species number, and species density among different regions (K1–K7) of Kenya.

## Results

### Families, genera and species of indigenous plants

A total of 6293 Kenyan indigenous vascular plants were recorded by FTEA, representing 225 families and 1538 genera, of which 95.5% are angiosperms, 4.0% are monilophytes, 0.37% are Lycophytes and a very small percentage are gymnosperms. Three families, 5 genera and 23 species belong to lycophytes; 27 families, 87 genera and 252 species belong to monilophytes; 3 families, 3 genera and 5 species belong to gymnosperms; 192 families, 1443 genera and 6013 species belong to angiosperms. Based on new systematic systems, the families number of SLFGKVP decrease to 224, including 3 families of Lycophytes, 28 families of monilophytes, 3 families of gymnosperms and 190 families of angiosperms. Because we fully accepted FTEA"s treatment on synonyms, the genera and species number kept the same.

The top three species-rich families of Kenyan indigenous vascular plants are Fabaceae, Poaceae and Asteraceae, which contain 576, 565 and 403 species, respectively. The top three species-rich genera are *Euphorbia* (Euphorbiaceae), *Cyperus* (Cyperaceae) and *Crotalaria* (Fabaceae), which contain 95, 94 and 93 species, respectively. The most species-rich family and genus of monilophytes are Aspleniaceae and *Asplenium* with 51 species (Table 1).

**Table 1.** The 13 largest families with more than 100 species, and the 12 largest genera with more than 50 species.

Family	Genera	Species	Genus	Species
Fabaceae	98	576	*Euphorbia* L. (Euphorbiaceae)	95
Poaceae	137	565	*Cyperus* L. (Cyperaceae)	94
Asteraceae	99	403	*Crotalaria* L. (Fabaceae)	93
Acanthaceae	42	279	*Indigofera* L. (Fabaceae)	70
Cyperaceae	29	274	*Ipomoea* L. (Convolvulaceae)	57
Rubiaceae	74	265	*Aloe* L. (Asphodelaceae)	55
Orchidaceae	47	243	*Plectranthus* L"Hér. (Lamiaceae)	54
Apocynaceae	70	235	*Justicia* L. (Acanthaceae)	54
Euphorbiaceae	29	219	*Vernonia* Schreb. (Asteraceae)	52
Malvaceae	40	219	*Asplenium* L. (Aspleniaceae)	51
Lamiaceae	32	206	*Commiphora* Jacq. (Burseraceae)	51
Convolvulaceae	20	118	*Eragrostis* Wolf (Poaceae)	50
Asparagaceae	13	104		

### Endemism

There is no endemic family in Kenya, but there is one endemic genus, which is *Dibrachionostylus*, a monotypic genus of Rubiaceae restricted to K4. The genus has only one species, *D. kaessneri* and is closely related to *Hedythyrsus* and *Agathisanthemum* (Verdcourt 1976).

In total, 467 endemic species including unnamed ones were recorded in FTEA. The endemic taxa of Lycophytes are mainly in *Isoetes* (Isoetaceae), 3 species out of 4 in this genus in Kenya being endemic. *Lycopodium aberdaricum* is also an endemic lycophyte only found in K3 and K4. There are only 4 endemic species in monilophytes and 3 belong to Marsileaceae. Another unnamed species of *Asplenium* (Aspleniaceae) recorded as endemic. There are no endemic gymnosperms. However, in angiosperms, 459 species are endemic, with the top contributing families for endemics the Euphorbiaceae (50 endemic species), Fabaceae (40 endemic species) and Acanthaceae (33 endemic species) (Table 2). There are 12 families with more than 10 endemic species, with the endemic ones in Asphodelaceae contributing 38.33% of the total species in that family (Table 2).

**Table 2.** The number of endemic plant species and total species of the top 12 families.

	Endemic species	Species in family	Endemism in family
Euphorbiaceae	50	219	22.83%
Fabaceae	40	576	6.94%
Acanthaceae	33	279	11.82%
Rubiaceae	29	265	10.94%
Asteraceae	29	403	7.19%
Asphodelaceae	23	60	38.33%
Apocynaceae	23	235	9.78%
Poaceae	18	565	3.19%
Lamiaceae	15	206	7.28%
Cyperaceae	12	274	4.38%
Vitaceae	12	63	19.05%
Cucurbitaceae	10	80	12.50%

### Exotic plants of Kenya

A total number of 588 exotic plants including 212 naturalized species of Kenya were recorded in FTEA, which belong to 62 families and 302 genera. The top 10 exotic families and their species number are shown in Table 3. Myrtaceae, Fabaceae, Asteraceae and Solanaceae are the 4 largest families which have more than 40 exotic species, and *Eucalyptus* (Myrtaceae) is the largest exotic genus with 99 exotic species.

**Table 3.** The number of exotic plant species of the top ten families in Kenya.

Family	Exotic species
Myrtaceae	133
Fabaceae	54
Asteraceae	44
Solanaceae	42
Bignoniaceae	25
Euphorbiaceae	25
Lamiaceae	24
Amaryllidaceae	22
Apocynaceae	19
Malvaceae	18

### Distribution patterns

**Figure 2.**
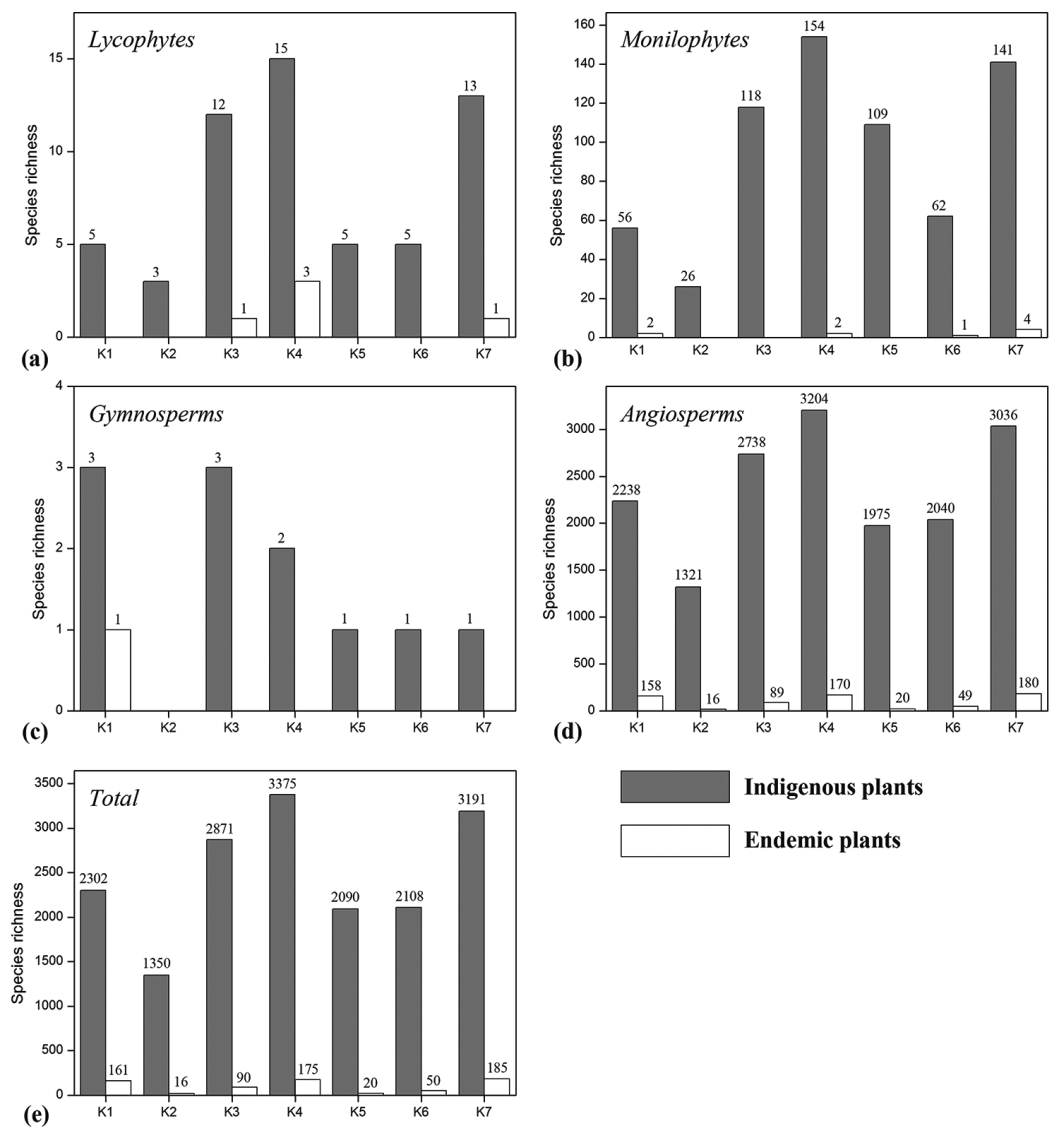
Lycophytes (**a**), monilophytes (**b**), gymnosperms (**c**), angiosperms (**d**) and total (**e**) species richness of indigenous (grey bars) and endemic (white bars) vascular plants in K1–K7 regions of Kenya.

The total number of indigenous and endemic species within each distribution region was counted (Fig. 2). Because species in gymnosperms are few, there is no significant uniformity (Fig. 2c). The results of Lycophytes (Fig. 2a), monilophytes (Fig. 2b), angiosperms (Fig. 2d) and the total species (Fig. 2e; Table 4) had similar patterns among seven regions and showed that K4 and K7 are the two most species-rich regions with regard to both total and endemic species. If the areas of different regions were taken into account, K3 and K5 have the highest density of total vascular plants, with ca. 776 species/10000 km^2^ in K3 and ca. 768 species/10000 km^2^ in K5. K3 and K7 have the highest density of endemic vascular plants, with ca. 24 species/10000 km^2^ in K3 and ca. 27 species/10000 km^2^ in K7. In contrast, K1 has the lowest density of total species (ca. 86 species/10000 km^2^) and K2 has the lowest density of endemic species (ca. 4 species/10000 km^2^) (Table 4).

## Discussion

### The diversity of Kenya vascular plants

Kenya has many unique geographical features resulting in the great diversity of plants. FTEA has recorded 6881 vascular plants in Kenya (including 588 exotic species), although the total species number is estimated to be 7000 to 8000 or more. The number of species is still increasing because of new records, new species, new cultivated and invasive plants. Recently, researchers from China and Kenya have found several new species from Kenya, such as *Sedum keniense* (Crassulaceae) (Zhou et al. 2016a), *Zehneria subcoraicea* (Cucurbitaceae) (Zhou et al. 2016b), *Z. longiflora* (Cucurbitaceae) (Wei et al. 2017), *Cissampelos keniensis* (Menispermaceae) (Zhou et al. 2017) and *Adenia angulosa* (Ngumbau et al. 2017).

The top three species-rich families of Kenya are Fabaceae, Poaceae and Asteraceae, which are also the largest families in the world, having 745 genera/19560 species, 707 genera/11337 species and 1620 genera/23600 species respectively (Stevens 2012). The proportion of Kenyan plants within the top total species in the world in these families is very small, though the endemism in them is relatively high (Table 1, 2).

Totally, 3375 indigenous vascular plants have been found in K4 (Fig. 2e; Table 4). The main vegetation types of K4 is Afromontane rain forest, Afromontane moist transitional forest, Dry *Combretum* wooded grassland and *Acacia-Commiphora* deciduous bushland-thicket (VECEA team 2015). The two most species-rich areas of K4 are Mt. Kenya and its neighboring Aberdare Ranges (Fig. 1). Montane forest vegetation as well as the unique Afroalpine vegetation also have high plant diversity and special species (Mittermeier et al. 2004, 2011, Gehrke and Linder 2014), such as *Vitex keniensis* (Lamiaceae), *Impatiens fischeri* (Balsaminaceae), *Cyphostemma grahamii* (Vataceae), *Ranunculus keniensis* (Ranunculaceae), *Dendrosenecio keniensis* (Asteraceae), *Lobelia deckenii* (Campanulaceae) (Young 1985, Bussmann 1995). There is also an endemic genus, *Dibrachionostylus* (Rubiaceae), recorded in K4 (Verdcourt 1976). There are at least 774 species, subspecies and varieties of vascular plants within the Aberdare Ranges, belonging to 213 genera, 128 families (Schmitt 1991). About 880 species, subspecies and varieties belonging to 479 genera and 146 families from Mt. Kenya below 3200 m altitude were recorded by Beentje (1991) and Bussman (1994), and a recent research results showed that more than 1500 vascular species are growing in this area, representing over 20% of total plants in Kenya (Zhou 2017). The Coastal Forests of Eastern Africa is one of the two diversity hotpots in east Africa, which is expanded to Somalia and Mozambique (Mittermeier et al. 2004, 2011). In our study, we recognise 3191 indigenous plants and 185 endemic plants in K7 (Fig. 2e; Table 4). K7 is located in the coastal areas of Kenya (Fig. 1) and has highest density of endemic plants with ca. 27 species/10000 km^2^, though the density of total indigenous plants is not very high with ca. 471 species/1000 km^2^ (Table 4). The two main vegetation types of K7 are *Acacia-Commiphora* deciduous bush land-thicket and Coastal mosaic (VECEA team 2015). A number of tropical coastal plants are living here, such as *Ceriops tagal* and *Bruguiera gymnorhiza* (Rhizophoraceae), *Sonneratia alba* (Lythraceae), *Xylocarpus granatum* (Meliaceae) and *Lumnitzera racemosa* (Combretaceae) (Dharani 2011).

**Table 4.** The number and density of total and endemic species in K1–K7 regions in Kenya.

Regions	Area/10000 km^2^	Total species	Endemic species	Density of total species	Density of endemic species
K1	26.79	2302	161	85.93	6.01
K2	4.55	1350	16	296.70	3.52
K3	3.7	2871	90	775.95	24.32
K4	9.29	3375	175	363.29	18.84
K5	2.72	2090	20	768.38	7.35
K6	4.44	2108	50	474.77	11.26
K7	6.77	3191	185	471.34	27.33

### Flora of Kenya

To date, there is no known regional flora within Kenya. "Flora of Tropical Africa", "Flora of East Tropical Africa" and other floras all have a long publication history. However, some information in them is not accurate, and a lot of species are still unnamed. In recent years, many new species, new records and new taxonomic treatments have been found and proposed. A series of surveys on plant resources at some biodiversity hotspots of Kenya have been carried out. Numerous plant checklists covering different regions of Kenya have been published, such as Mt. Elgon (Tweedie 1976), Mt. Nyiru (Bytebier and Bussmann 2000), Taita hills (Thijs et al. 2013), Nandi Forests (Girma et al. 2015). Therefore, an up-to-date and comprehensive flora (i.e. "Flora of Kenya") is urgently needed, as this will further promote further botanical studies and help action conservation measures to protect the plant diversity in Kenya. At present, Chinese researchers are actively involved in the preparation of a new Flora of Kenya, and contributing their skills to help the Kenyan people determine exactly what plants live in Kenya, to enable better protection for them, and to develop and utilize where practical the extremely high diversity of Kenyan plants.

### Conservation of plant diversity in Kenya

Due to human disturbance and destruction, exotic plant invasion, climate change, deterioration of ecological environment and other factors, the plant diversity of Kenya is also facing severe pressures (FAO 1981, Barnes 1990, Bussmann 1996, Gathaara 1999). For example, at the foot of Mt. Kenya, a large area of the original forest was cut down for plantations of *Cupressus lusitanica*, *Pinus patula* and *Eucalyptus* spp. for timber and also some cultivations of crops such as banana (*Musa* spp.), potato (*Solanum tuberosum*) and maize (*Zea mays*) (Bussmann 1996, Gathaara 1999). K3, K4, K5 and K7 have high plant diversity and high density of total and endemic plants. Consideration of better protection measures of a representation of these distribution regions is urgently needed.

## Supplementary materials

Supplementary material 1 The Synoptic List of Families and Genera of Kenyan Vascular Plants (SLFGKVP) Data type: species data Explanation note: There are 6293 indigenous and 588 exotic vascular plants of Kenya in total, which belong to 1752 genera and 223 families. Families of Lycophytes and monilophytes are arranged by PPG I system (PPG I 2016), families of gymnosperms are arranged by Christenhusz gymnosperms system (Christenhusz et al. 2011a), and families of angiosperms are arranged by APG IV system (APG IV 2016). This dataset is made available under the Open Database License (http://opendatacommons.org/licenses/odbl/1.0/). The Open Database License (ODbL) is a license agreement intended to allow users to freely share, modify, and use this Dataset while maintaining this same freedom for others, provided that the original source and author(s) are credited.Yadong Zhou, Bing Liu, Yuvenlis Mbuni, Xue Yan, Geoffrey Mwachala, Gugangwan Hu, Qingfeng Wang

## References

[B1] Agnew ADQ Agnew S (1994) Upland Kenya Wild Flowers, Second Edition. East Africa Natrual History Society, Nairobi, Kenya.

[B2] Agnew ADQ (1974) Upland Kenya Wild Flowers. Oxford University Press, London, UK.

[B3] Agnew ADQ (2013) Upland Kenya Wild Flowers and Ferns, Third Edition. Nature Kenya Publications, Nairobi, Kenya.

[B4] APG (1998) An ordinal classification for the families of flowering plants. Annals of the Missouri Botanical Garden 85: 531–553. https://doi.org/10.2307/2992015

[B5] APG II (2003) An update of the Angiosperm Phylogeny Group classification for the orders and families of flowering plants: APG II. Botanical Journal of the Linnean Society 141: 399–436. https://doi.org/10.1046/j.1095-8339.2003.t01-1-00158.x

[B6] APG III (2009) An update of the Angiosperm Phylogeny Group classification for the orders and families of flowering plants: APG III. Botanical Journal of the Linnean Society 161: 105–121. https://doi.org/10.1111/j.1095-8339.2009.00996.x

[B7] APG IV (2016) An update of the Angiosperm Phylogeny Group classification for the orders and families of flowering plants: APG IV. Botanical journal of the Linnean Society 181(1): 1–20. https://doi.org/10.1111/boj.12385

[B8] Beentje H (1991) Forests of Mount Kenya—vegetation and human uses. In: Ojany FF Lusigi W Rheker JR Taiti SW Wiesmann U Winiger M (Eds) Proceedings of the International Workshop on Ecology and Socio-Economy of Mount Kenya Area, Nanyuki. University of Bern, Swiss, 47–57.

[B9] Beentje HJ (1994) Kenya trees, shrubs and lianas. National Museums of Kenya, Nairobi, Kenya.

[B10] Blundell M (1982) The wild flowers of Kenya. Collins, London, UK.

[B11] Blundell M (1987) Collins guide to the wild flowers of East Africa. Collins, London, UK.

[B12] Bussmann RW Beck E (1995) The forests of Mt. Kenya (Kenya), a phytosociological synopsis. Phytocoenologia 25(4): 467–560. https://doi.org/10.1127/phyto/25/1995/467

[B13] Bussmann RW (1994) The forests of Mount Kenya (Kenya): vegetation, ecology, destruction and management of a tropical mountain forest ecosystem. University of Bayreuth, Bayreuth.

[B14] Bussmann RW (1996) Destruction and Management of Mount Kenya"s Forests. Ambio, 25(5): 314–317.

[B15] Bytebier B Bussmann RW (2000) The Vegetation of Mount Nyiru (Samburu District, Kenya) A Checklist and Syntaxonomical Survey. Journal of East African Natural History 89(1): 45–71. https://doi.org/10.2982/0012-8317(2000)89[45:TVOMNS]2.0.CO;2

[B16] Cheng WC Fu LK (1978) Fl. Reipubl. Popularis Sin. 7, Gymnospermae. Science Press, Beijing.

[B17] Ching JC (1987a) Chinese fern families and genera: systematic arrangement and historical origin. Acta Phytotax. Sin. 16(3): 1–19.

[B18] Ching JC (1987b) Chinese fern families and genera: systematic arrangement and historical origin. Acta Phytotax. Sin. 16(4): 16–37.

[B19] Christenhusz MJM Reveal JL Farjon A Gardner MF Mill RR Chase MW (2011b) A new classification and linear sequence of extant gymnosperms. Phytotaxa 19: 55–70. https://doi.org/10.11646/phytotaxa.19.1.3

[B20] Christenhusz MJM Zhang XC Schneider H (2011) A linear sequence of extant families and genera of lycophytes and ferns. Phytotaxa 19: 7–54. https://doi.org/10.11646/phytotaxa.19.1.2

[B21] Dale IR Greenway PJ (1961) Kenya trees and shrubs. Buchanan"s Kenya Estates Ltd., Nairobi.

[B22] Daniel et al. (1868–1933) Flora of Tropical Africa. L. Reeve & Co., London.

[B23] Dharani N (2006) Field Guide to Acacis of East Africa. Struik Nature, Cape Town, South Africa.

[B24] Dharani N (2011) Field Guide to Common Trees and Shrubs of East Africa, Second Edition. Struik Nature, Cape Town.

[B25] Dharani N Yenesew A (2010) Medicinal Plants of East Africa-An Illustrated Guide. University of Nairobi Press, Nairobi.

[B26] Edwards DC (1940) A Vegetation Map of Kenya with Particular Reference to Grassland Types. Journal of Ecology 28(2): 377–385. https://doi.org/10.2307/2256235

[B27] FAO (1981) Tropical forest resource asessment plan – forest resources of Tropical Africa. Technical Report 2, Rome.

[B28] FTEA (1952–2012) Flora of Tropical East Africa. Royal Botanic Gardens, Kew, London.

[B29] Gao JY (2004) Guide to the world stattes: Kenya. Social Sciences Academic Press, Beijing.

[B30] Gathaara GN (1999) Aerial survey of the destruction of Mt. Kenya, Imenti and Ngare Ndare forest reserves. Kenya Wildlife Service, Nairobi.

[B31] Gehrke B Linder HP (2014) Species richness, endemism and species composition in the tropical Afroalpine flora. Alpine Botany 124: 165–177. https://doi.org/10.1007/s00035-014-0132-0

[B32] Girma A Fischer E Dumbo B (2015) Vascular Plant Diversity and Community Structure of Nandi Forests, Western Kenya. Journal of East African Natural History 103(2): 125–152. https://doi.org/10.2982/028.103.0202

[B33] Ibrahim KM Kabuye CHS (1987) An illustrated manual of Kenya Grasses. FAO, Rome, Italy.

[B34] Karmali J (1988) The beautiful plants of Kenya. Text Book Centre Ltd, Nairobi.

[B35] Kokwaro JO (2009) Medicinal Plants of East Africa (3^rd^ edn). University of Nairobi Press, Nairobi.

[B36] Liu B Ye JF Yang Y Lai YJ Zeng G Lin QW (2015) Families and genera of Chinese angiosperms: a synoptic classification based on APG III. Biodiversity Science 23(2): 225–231. https://doi.org/10.17520/biods.2015052

[B37] Liu HM Jiang RH Guo J Hovenkamp P Perrie LR Shepherd L Hennequin S Schneider H (2013) Towards a phylogenetic classification of the climbing fern genus Arthropteris. Taxon 62(62): 688–700. https://doi.org/10.12705/624.26

[B38] Liu HM Zhang XC Zeng H (2009) Application of DNA Sequences in Pteridophyte Phylogenetic Study. Chinese Bulletin of Botany 44(2): 143–158.

[B39] MU (2009) Mount Kenya National Park and National Forest, Kenya. http://www.eoearth.org/view/article/154706

[B40] Maundu P Tengnas B (2005) Useful trees and shrubs for Kenya. ICRAF-ECA, Nairobi.

[B41] Ngumbau VM Nyange M Dai C Zhong ZX Wei N Malombe I Hu GW Wang QF (2017) Adenia angulosa (Passifloraceae), a new species from coastal forests of Kenya and Tanzania. Phytotaxa 313(1): 137–142. https://doi.org/10.11646/phytotaxa.313.1.10

[B42] Ojany FF Ogendo RB (1973) Kenya: A Study in Physical and Human Geography. Longman, Nairobi.

[B43] Orodho AB (2006) Country Pasture/Forage Resource Profiles: Kenya. http://www.fao.org/ag/AGP/AGPC/doc/Counprof/Kenya.htm.

[B44] Polhill D (1988) Flora of Tropical East Africa: Index of Collection Localities. Royal Botanic Gardens, Kew, London.

[B45] PPG I (2016) A community-derived classification for extant lycophytes and ferns. Journal of Systematics and Evolution 54(6): 563–603. https://doi.org/10.1111/jse.12229

[B46] Pryer KM Schuettpelz E Wolf PG Granfill R (2004) Phylogeny and evolution of ferns (Monilophytes) with a focus on the early Leptosporangiate divergences. American Journal of Botany 91(10): 1582–1598. https://doi.org/10.3732/ajb.91.10.1582 21652310 10.3732/ajb.91.10.1582

[B47] Schmitt K (1991) The Vegetation of the Aberdare National Park, Kenya. Hochgebirgsforschung 8: 1–259.

[B48] Simpson MG (2010) Plant Systematics (2^nd^ edn). Academic Press, New York. https://doi.org/10.1016/B978-0-12-374380-0.50001-4

[B49] Singh G (2010) Plant systematics, an integrated approach (3^rd^ edn). CRC Press, Boca Raton. https://doi.org/10.1201/b10255

[B50] Smith AR Pryer KM Schuettpelz E Korall P Schneider H Wolf PG (2006) A classification for extant ferns. Taxon 55(3): 705–731. https://doi.org/10.2307/25065646

[B51] Sombroek WC Braun HMH van der Pouw BJA (1982) Explanatory soil map and agro-climatic zone map of Kenya. Report E1. National Agricultural Laboratories, Soil Survey Unit, Nairobi, 56 pp.

[B52] Speck H (1982) Soils of the Mount Kenya Area: Their Formation, Ecological, and Agricultural Significance. Mountain Research and Development 2(2): 201–221. https://doi.org/10.2307/3672965

[B53] Stevens PF (2012) Angiosperm Phylogeny Website. http://www.mobot.org/MOBOT/research/APweb/

[B54] Stewart J (2003) Orchids of Kenya. Timber Press, Portland.

[B55] Thijs KW Roelen I Musila WM (2013) Field Guide to the Woody Plants of Taita Hills, Kenya. Journal of East African Natural History 102(1, 2): 1–272.

[B56] Tweedie EM (1976) Habitats and Check-List of Plants on the Kenya Side of Mount Elgon. Kew Bulletin 31(2): 227–257. https://doi.org/10.2307/4109170

[B57] VECEA team (2015) Potential natural vegetation of eastern Africa, Version 2.0. http://vegetationmap4africa.org/Home.html

[B58] Verdcourt B (1976) Rubiaceae. In: Polhill RM (Ed.) Flora of Tropical East Africa. Royal Botanic Gardens, Kew, London.

[B59] Wang QF Zhou YD Hu GW Yan X Mwahcala GM Gituru RW (2016) Common Plants of Kenya. Hubei Science & Technology Press, Wuhan.

[B60] Wei N Miyawa DO David MK Ngumbau VM Zhong ZX Mawachla G Hu GW Wang QF (2017) *Zehneria longiflora* (Cucurbitaceae), a new species from Kenya. Phytotaxa 324(1): 89–94. https://doi.org/10.11646/phytotaxa.324.1.7

[B61] Yang Y Fu DZ Wang Q (2004) Origin of flowers: hypotheses and evidence. Acta Botanica Boreali-Occidentalia Sinica 24(12): 2366–2380.

[B62] Young TP Peacock MM (1985) Vegetative key to the alpine vascular plants of Mount Kenya. Journal of the East Africa National History Society and National Museum 75(185): 1–9.

[B63] Zhang LB Zhang L (2015) Didymochlaenaceae: A new fern family of eupolypods I (Polypodiales). Taxon 64(1): 27–38. https://doi.org/10.12705/641.4

[B64] Zhou YD Hu GW Yan X Mwachala G Gituru RW Wang QF (2016a) *Sedum keniense* (Crassulaceae), a new species from Mt. Kenya, East Africa. Phytotaxa 261(2): 177–184. https://doi.org/10.11646/phytotaxa.261.2.7

[B65] Zhou YD Mbuni Y Hu GW Yan X Mwachala G Wang QF (2016b) *Zehneria subcoriacea* (Cucurbitaceae), a New Species from Kenya. Phytotaxa 277(3): 282–286. https://doi.org/10.11646/phytotaxa.277.3.6

[B66] Zhou YD (2017) Inventory and Diversity of Vascular Plants in Mt. Kenya, East Africa. PhD Thesis, Wuhan Botanical Garden, Wuhan.

[B67] Zhou YD Mbuni Y Hu GW Zhong ZX Yan X Mwachala G Wang QF (2017) *Cissampelos keniensis* (Menispermaceae), a new species from Mt. Kenya, East Africa. Phytotaxa 292(1): 57–64. https://doi.org/10.11646/phytotaxa.292.1.5

