# Supplementary material for: Vascular flora of Kenya, based on the Flora of Tropical East Africa
Source: PhytoKeys. 2017 Nov 16;(90):113–26. doi: 10.3897/phytokeys.90.20531 (PMC5740400; doi:10.3897/phytokeys.90.20531)
Supplement: Supplementary material 1 — The Synoptic List of Families and Genera of Kenyan Vascular Plants (SLFGKVP) [file phytokeys-90-113-s001.doc]

**Appendix I The Synoptic List of Families and Genera of Kenyan Vascular Plants (SLFGKVP).**

Yadong Zhou, Bing Liu, Yuvenlis Mbuni, Xue Yan, Geoffrey Mwachala, Gugangwan Hu, Qingfeng Wang

There are 6293 indigenous and 588 exotic vascular plants of Kenya in total, which belong to 1752 genera and 223 families. Families of Lycophytes and monilophytes are arranged by PPG I system (PPG I 2016), families of gymnosperms are arranged by Christenhusz gymnosperms system (Christenhusz et al. 2011a), and families of angiosperms are arranged by APG IV system (APG IV 2016). Genera of each family were arranged by alphabetical order.

|  | **Indigenous (Exotic) species** | **FTEA Family** |
| --- | --- | --- |
| **Lycophytes** | **23** |  |
| **Lycopodiales** |  |  |
| f1. **Lycopodiaceae** |  |  |
| *Huperzia* Bernh. | 6 | Lycopodiaceae |
| *Lycopodiella* Holub | 1 | Lycopodiaceae |
| *Lycopodium* L. | 2 | Lycopodiaceae |
| **Isoetales** |  |  |
| f2. **Isoetaceae** |  |  |
| *Isoetes* L. | 4 | Isoetaceae |
| **Selaginellales** |  |  |
| f3. **Selaginellaceae** |  |  |
| *Selaginella* P. Beauv. | 10 | Selaginellaceae |
| **Monilophytes** | **252 (1)** |  |
| **Equisetales** |  |  |
| f4. **Equisetaceae** |  |  |
| *Equisetum* L. | 1 | Equisetaceae |
| **Psilotales** |  |  |
| f5. **Psilotaceae** |  |  |
| *Psilotum* Sw. | 1 | Psilotaceae |
| **Ophioglossales** |  |  |
| f6. **Ophioglossaceae** |  |  |
| *Ophioglossum* L. | 10 | Ophioglossaceae |
| **Marattiales** |  |  |
| f7. **Marattiaceae** |  |  |
| *Marattia* Sw. | 1 | Marattiaceae |
| **Osmundales** |  |  |
| f8. **Osmundaceae** |  |  |
| *Osmunda* L. | 1 | Osmundaceae |
| **Hymenophyllales** |  |  |
| f9. **Hymenophyllaceae** |  |  |
| *Abrodictyum* C. Presl | 1 | Hymenophyllaceae |
| *Crepidomanes* C. Presl | 4 | Hymenophyllaceae |
| *Didymoglossum* Desv. | 1 | Hymenophyllaceae |
| *Hymenophyllum* Sm. | 5 | Hymenophyllaceae |
| *Polyphlebium* Copel. | 1 | Hymenophyllaceae |
| *Vandenboschia* Copel. | 1 | Hymenophyllaceae |
| **Gleicheniales** |  |  |
| f10. **Gleicheniaceae** |  |  |
| *Gleichenella* Ching | 1 | Gleicheniaceae |
| *Dicranopteris* Bernh. | 1 | Gleicheniaceae |
| **Schizaeales** |  |  |
| f11. **Lygodiaceae** |  |  |
| *Lygodium* Sw. | 2 | Schizaeaceae |
| f12. **Anemiaceae** |  |  |
| *Anemia* Sw. | 1 | Schizaeaceae |
| *Mohria* Sw. | 1 | Schizaeaceae |
| **Salviniales** |  |  |
| f13. **Salviniaceae** |  |  |
| *Azolla* Lam. | 2 | Azollaceae |
| f14. **Marsileaceae** |  |  |
| *Marsilea* L. | 9 | Marsileaceae |
| **Cyatheales** |  |  |
| f15. **Cyatheaceae** |  |  |
| *Cyathea* Sm. | 2 | Cyatheaceae |
| **Polypodiales** |  |  |
| f16. **Pteridaceae** |  |  |
| *Acrostichum* L. | 1 | Pteridaceae |
| *Actiniopteris* Link | 3 | Actiniopteridaceae |
| *Adiantum* L. | 8 | Adiantaceae |
| *Anogramma* Link | 1 | Adiantaceae |
| *Antrophyum* Kaulf. | 1 | Vittariaceae |
| *Aspidotis* (Nutt. ex Hook.) Copel. | 1 | Adiantaceae |
| *Ceratopteris* Brongn. | 1 | Parkeriaceae |
| *Cheilanthes* Sw. | 5 | Adiantaceae |
| *Coniogramme* Fée | 1 | Adiantaceae |
| *Doryopteris* J. Sm. | 1 | Adiantaceae |
| *Negripteris* Pic. Serm. | 1 | Adiantaceae |
| *Notholaena* R. Br. | 1 | Adiantaceae |
| *Pellaea* Link | 8 | Adiantaceae |
| *Pityrogramma* Link | 2 | Adiantaceae |
| *Pteris* L. | 14 | Pteridaceae |
| *Vittaria* Sm. | 4 | Vittariaceae |
| f17. **Dennstaedtiaceae** |  |  |
| *Blotiella* R.M. Tryon | 3 | Dennstaedtiaceae |
| *Histiopteris* (J. Agardh) J. Sm. | 1 | Dennstaedtiaceae |
| *Hypolepis* Bernh. | 2 | Dennstaedtiaceae |
| *Microlepia* C. Presl | 1 | Dennstaedtiaceae |
| *Pteridium* Gled. ex Scop. | 1 | Dennstaedtiaceae |
| f18. **Cystopteridaceae** |  |  |
| *Cystopteris* Bernh. | 1 | Woodsiaceae |
| f19. **Aspleniaceae** |  |  |
| *Asplenium* L. | 51 | Aspleniaceae |
| *Ceterach* Willd. | 1 | Aspleniaceae |
| f20. **Blechnaceae** |  |  |
| *Blechnum* L. | 4 | Blechnaceae |
| *Stenochlaena* J. Sm. | 1 | Blechnaceae |
| f21. **Athyriaceae** |  |  |
| *Athyrium* Roth | 2 | Woodsiaceae |
| *Diplazium* Sw. | 4 | Woodsiaceae |
| *Dryoathyrium* Ching | 1 | Woodsiaceae |
| f22. **Thelypteridaceae** |  |  |
| *Amauropelta* Kunze | 2 | Thelypteridaceae |
| *Ampelopteris* Kunze | 1 | Thelypteridaceae |
| *Amphineuron* Holttum | 1 | Thelypteridaceae |
| *Christella* H.Lév. | 7 | Thelypteridaceae |
| *Cyclosorus* Link | 1 | Thelypteridaceae |
| *Pneumatopteris* Nakai | 2 | Thelypteridaceae |
| *Pseudocyclosorus* Ching | 1 | Thelypteridaceae |
| *Pseudophegopteris* Ching | 2 | Thelypteridaceae |
| *Stegnogramma* Blume | 1 | Thelypteridaceae |
| *Thelypteris* Schmidel | 1 | Thelypteridaceae |
| f23. **Didymochlaenaceae** |  |  |
| *Didymochlaena* Desv. | 1 | Dryopteridaceae |
| f24. **Hypodematiaceae** |  |  |
| *Hypodematium* Kunze | 1 | Dryopteridaceae |
| f25. **Dryopteridaceae** |  |  |
| *Arachniodes* Blume | 1 | Dryopteridaceae |
| *Bolbitis* Schott | 2 | Lomariopsidaceae |
| *Ctenitis* (C. Chr.) C. Chr. | 1 | Dryopteridaceae |
| *Cyrtomium* C. Presl | 1 | Dryopteridaceae |
| *Dryopteris* Adans. | 11 | Dryopteridaceae |
| *Elaphoglossum* Schott ex J. Sm. | 10 | Lomariopsidaceae |
| *Megalastrum* Holttum | 1 | Dryopteridaceae |
| *Nothoperanema* (Tagawa) Ching | 1 | Dryopteridaceae |
| *Polystichum* Roth | 4 | Dryopteridaceae |
| f26. **Nephrolepidaceae** |  |  |
| *Nephrolepis* Schott | 3 (1) | Oleandraceae |
| f27. **Lomariopsidaceae** |  |  |
| *Lomariopsis* Fée | 1 | Lomariopsidaceae |
| f28. **Tectariaceae** |  |  |
| *Arthropteris* J. Sm. | 2 | Oleandraceae |
| *Tectaria* Cav. | 3 | Dryopteridaceae |
| f29. **Oleandraceae** |  |  |
| *Oleandra* Cav. | 1 | Oleandraceae |
| f30. **Davalliaceae** |  |  |
| *Davallia* Sm. | 1 | Davalliaceae |
| f31. **Polypodiaceae** |  |  |
| *Drynaria* (Bory) J. Sm. | 2 | Polypodiaceae |
| *Grammitis* Sw. | 2 | Grammitidaceae |
| *Lellingeria* A.R. Sm. & R.C. Moran | 1 | Grammitidaceae |
| *Lepisorus* (J. Sm.) Ching | 2 | Polypodiaceae |
| *Loxogramme* (Blume) C. Presl | 1 | Polypodiaceae |
| *Melpomene* A.R. Sm. & R.C. Moran | 1 | Grammitidaceae |
| *Microgramma* C. Presl | 1 | Polypodiaceae |
| *Microsorum* Link | 1 | Polypodiaceae |
| *Phymatosorus* Pic. Serm. | 1 | Polypodiaceae |
| *Platycerium* Desv. | 2 | Polypodiaceae |
| *Pleopeltis* Humb. & Bonpl. ex Willd. | 1 | Polypodiaceae |
| *Pyrrosia* Mirb. | 1 | Polypodiaceae |
| **Gymnosperms** | **5** |  |
| **Cycadales** |  |  |
| f32. **Zamiaceae** |  |  |
| *Encephalartos* Lehm. | 2 | Cycadaceae |
| **Araucariales** |  |  |
| f33. **Podocarpaceae** |  |  |
| *Podocarpus* L'Hér. ex Pers. | 2 | Podocarpaceae |
| **Cupressales** |  |  |
| f34. **Cupressaceae** |  |  |
| *Juniperus* L. | 1 | Cupressaceae |
| **Angiosperms** | **6013 (587)** |  |
| **Nymphaeales** |  |  |
| f35. **Cabombaceae** |  |  |
| *Brasenia* Schreb. | 1 | Cabombaceae |
| f36. **Nymphaeaceae** |  |  |
| *Nymphaea* L. | 2 | Nymphaeaceae |
| **Canellales** |  |  |
| f37. **Canellaceae** |  |  |
| *Warburgia* Engl. | 2 | Canellaceae |
| **Piperales** |  |  |
| f38. **Piperaceae** |  |  |
| *Peperomia* Ruiz & Pav. | 9 | Piperaceae |
| *Piper* L. | 3 | Piperaceae |
| f39. **Aristolochiaceae** |  |  |
| *Aristolochia* L. | 3 | Aristolochiaceae |
| *Hydnora* Thunb. | 2 | Hydnoraceae |
| **Magnoliales** |  |  |
| f40. **Myristicaceae** |  |  |
| *Cephalosphaera* Warb. | 1 | Myristicaceae |
| f41. **Annonaceae** |  |  |
| *Annona* L. | 1 | Annonaceae |
| *Artabotrys* R. Br. | 2 (1) | Annonaceae |
| *Asteranthe* Engler & Diels in Engler | 1 | Annonaceae |
| *Cananga* (DC.) Hook. f. & Thomson | 1 | Annonaceae |
| *Lettowianthus* Diels | 1 | Annonaceae |
| *Melodorum* Lour. | 1 | Annonaceae |
| *Mkilua* Verdc. | 1 | Annonaceae |
| *Monanthotaxis* Baill. | 5 | Annonaceae |
| *Monodora* Dunal | 2 | Annonaceae |
| *Ophrypetalum* Diels | 1 | Annonaceae |
| *Polyalthia* Blume | 1 | Annonaceae |
| *Uvaria* L. | 8 | Annonaceae |
| *Uvariodendron* (Engl. & Diels) R.E. Fr. | 3 | Annonaceae |
| *Uvariopsis* Engl. | 1 | Annonaceae |
| *Xylopia* L. | 2 | Annonaceae |
| **Laurales** |  |  |
| f42. **Hernandiaceae** |  |  |
| *Gyrocarpus* Jacq. | 2 | Hernandiaceae |
| *Hernandia* L. | 1 | Hernandiaceae |
| *Illigera* Blume | 1 | Hernandiaceae |
| f43. **Monimiaceae** |  |  |
| *Xymalos* Baill. ex Warb. | 1 | Monimiaceae |
| f44. **Lauraceae** |  |  |
| *Cassytha* Osbeck | 1 | Lauraceae |
| *Cinnamomum* Schaeff. | (1) | Lauraceae |
| *Laurus* L. | (1) | Lauraceae |
| *Ocotea* Aubl. | 2 | Lauraceae |
| *Persea* Mill. | (1) | Lauraceae |
| **Alismatales** |  |  |
| f45. **Araceae** |  |  |
| *Alocasia* (Schott) G. Don in Sweet | (1) | Araceae |
| *Amorphophallus* Blume ex Decne. | 4 (1) | Araceae |
| *Anchomanes* Schott | 1 | Araceae |
| *Anthurium* Schott | (1) | Araceae |
| *Arisaema* Mart. | 2 | Araceae |
| *Caladium* Vent. | (1) | Araceae |
| *Callopsis* Engl. | 1 | Araceae |
| *Colocasia* Schott | (1) | Araceae |
| *Culcasia* P. Beauv. | 2 | Araceae |
| *Dieffenbachia* Schott | (1) | Araceae |
| *Epipremnum* Schott | (1) | Araceae |
| *Gonatopus* Hook. f. ex Engl. | 1 | Araceae |
| *Lemna* L. | 4 | Lemnaceae |
| *Monstera* Adans. | (2) | Araceae |
| *Pistia* L. | 1 | Araceae |
| *Pseudowolffia* Hartog & Plas | 1 | Lemnaceae |
| *Sauromatum* Schott | 1 | Araceae |
| *Spirodela* Schleid. | 1 | Lemnaceae |
| *Stylochaeton* Lepr. (*Stylochiton* Schott of FTEA) | 3 | Araceae |
| *Typhonium* Schott | (1) | Araceae |
| *Wolffia* Horkel ex Schleid. | 1 | Lemnaceae |
| *Xanthosoma* Schott | (1) | Araceae |
| *Zamioculcas* Schott | 1 | Araceae |
| f46. **Alismataceae** |  |  |
| *Alisma* L. | 1 | Alismataceae |
| *Burnatia* Micheli in A. DC. & C. de Candolle | 1 | Alismataceae |
| *Limnophyton* Miq. | 1 | Alismataceae |
| f47. **Hydrocharitaceae** |  |  |
| *Egeria* Planch. | 1 | Hydrocharitaceae |
| *Enhalus* Rich. | 1 | Hydrocharitaceae |
| *Halophila* Thouars | 3 | Hydrocharitaceae |
| *Hydrilla* Rich. | 1 | Hydrocharitaceae |
| *Lagarosiphon* Harv. | 3 | Hydrocharitaceae |
| *Najas* L. | 3 | Najadaceae |
| *Ottelia* Pers. | 3 | Hydrocharitaceae |
| *Thalassia* Banks ex K.D. Koenig | 1 | Hydrocharitaceae |
| *Vallisneria* L. | 1 | Hydrocharitaceae |
| f48. **Aponogetonaceae** |  |  |
| *Aponogeton* L. f. | 6 | Aponogetonaceae |
| f49. **Zosteraceae** |  |  |
| *Zostera* L. | 1 | Zosteraceae |
| f50. **Potamogetonaceae** |  |  |
| *Potamogeton* L. | 6 | Potamogetonaceae |
| **Dioscoreales** |  |  |
| f51. **Burmanniaceae** |  |  |
| *Gymnosiphon* Blume | 1 | Burmanniaceae |
| f52. **Dioscoreaceae** |  |  |
| *Dioscorea* L. | 8 | Dioscoreaceae |
| *Tacca* J.R. Forst. & G. Forst. | 1 | Taccaceae |
| **Pandanales** |  |  |
| f53. **Velloziaceae** |  |  |
| *Xerophyta* Juss. | 2 | Velloziaceae |
| f54. **Pandanaceae** |  |  |
| *Pandanus* Parkinson | 3 | Pandanaceae |
| **Liliales** |  |  |
| f55. **Colchicaceae** |  |  |
| *Androcymbium* Willd. | 1 | Colchicaceae |
| *Gloriosa* L. | 1 | Colchicaceae |
| *Iphigenia* Kunth | 2 | Colchicaceae |
| *Wurmbea Thunb.* | 1 | Colchicaceae |
| f56. **Smilacaceae** |  |  |
| *Smilax* L. | 2 | Smilacaceae |
| f57. **Liliaceae** |  |  |
| *Lilium* L. | 1 | Liliaceae |
| **Asparagales** |  |  |
| f58. **Orchidaceae** |  |  |
| *Acampe* Lindl. | 1 | Orchidaceae |
| *Aerangis* Rchb. f. | 10 | Orchidaceae |
| *Angraecopsis* Kraenzl. | 3 | Orchidaceae |
| *Angraecum* Bory | 12 | Orchidaceae |
| *Ansellia* Lindl. | 1 | Orchidaceae |
| *Bolusiella* Schltr. | 2 | Orchidaceae |
| *Bonatea* Willd. | 4 | Orchidaceae |
| *Brachycorythis* Lindl. | 6 | Orchidaceae |
| *Brownleea* Harv. ex Lindl. | 1 | Orchidaceae |
| *Bulbophyllum* Thouars | 9 | Orchidaceae |
| *Calanthe* R. Br. | 1 | Orchidaceae |
| *Calyptrochilum* Kraenzl. | 1 | Orchidaceae |
| *Chamaeangis* Schltr. | 3 | Orchidaceae |
| *Chaseella* Summerh. | 1 | Orchidaceae |
| *Cheirostylis* Blume | 1 | Orchidaceae |
| *Cribbia* Senghas | 1 | Orchidaceae |
| *Cynorkis* Thou. | 3 | Orchidaceae |
| *Cyrtorchis* Schltr. | 2 | Orchidaceae |
| *Diaphananthe* Schltr. | 8 | Orchidaceae |
| *Disa* P. J. Bergius | 8 | Orchidaceae |
| *Disperis* Sw. | 7 | Orchidaceae |
| *Epipactis* Zinn | 1 | Orchidaceae |
| *Epipogium* Gmelin ex Borkhausen | 1 | Orchidaceae |
| *Eulophia* R. Br. ex Lindl. | 27 | Orchidaceae |
| *Habenaria* Willd. | 43 | Orchidaceae |
| *Holothrix* Rich. ex Lindl. | 5 | Orchidaceae |
| *Jumellea* Schltr. | 2 | Orchidaceae |
| *Liparis* Rich. | 2 | Orchidaceae |
| *Malaxis* Sol. ex Sw. | 1 | Orchidaceae |
| *Margelliantha* P. J. Cribb | 1 | Orchidaceae |
| *Microcoelia* Lindl. | 8 | Orchidaceae |
| *Nephrangis* Summerh. | 1 | Orchidaceae |
| *Nervilia* Comm. ex Gaudich. | 1 | Orchidaceae |
| *Oeceoclades* Lindl. | 2 | Orchidaceae |
| *Platycoryne* Rchb. f. | 1 | Orchidaceae |
| *Polystachya* Hook. | 30 | Orchidaceae |
| *Pteroglossaspis* Rchb. f. | 2 | Orchidaceae |
| *Rangaeris* (Schltr.) Summerh. | 2 | Orchidaceae |
| *Roeperocharis* Rchb. f. | 1 | Orchidaceae |
| *Satyrium* Sw. | 10 | Orchidaceae |
| *Solenangis* Schltr. | 3 | Orchidaceae |
| *Stolzia* Schltr. | 1 | Orchidaceae |
| *Triceratorhynchus* Summerh. | 1 | Orchidaceae |
| *Tridactyle* Schltr. | 8 | Orchidaceae |
| *Vanilla* Plum. ex Mill. | 2 | Orchidaceae |
| *Ypsilopus* Summerh. | 1 | Orchidaceae |
| *Zeuxine* Lindl. | 1 | Orchidaceae |
| f59. **Hypoxidaceae** |  |  |
| *Curculigo* Gaertn. | 1 | Hypoxidaceae |
| *Hypoxis* L. | 9 | Hypoxidaceae |
| *Molineria* Colla | (1) | Hypoxidaceae |
| f60. **Iridaceae** |  |  |
| *Aristea* Aiton | 3 | Iridaceae |
| *Dierama* K. Koch | 1 | Iridaceae |
| *Dietes* Salisb. | 1 | Iridaceae |
| *Freesia* Eckl. ex Klatt | 1 | Iridaceae |
| *Gladiolus* L. | 10 | Iridaceae |
| *Hesperantha* Ker Gawl. | 1 | Iridaceae |
| *Lapeirousia* Pourr. | 1 | Iridaceae |
| *Moraea* Mill. | 2 | Iridaceae |
| *Romulea* Maratti | 3 | Iridaceae |
| f61. **Asphodelaceae** |  |  |
| *Aloe* L. | 55 (2) | Aloaceae |
| *Bulbine* Wolf | 1 | Asphodelaceae |
| *Jodrellia* Baijnath | 1 | Asphodelaceae |
| *Kniphofia* Moench | 2 | Asphodelaceae |
| *Trachyandra* Kunth | 1 | Asphodelaceae |
| f62. **Amaryllidaceae** |  |  |
| *Allium* L. | (6) | Alliaceae |
| *Amaryllis* L. | (1) | Amaryllidaceae |
| *Ammocharis* Herb. | 2 | Amaryllidaceae |
| *Boophone* Herb. | 1 | Amaryllidaceae |
| *Clivia* Lindl. | (1) | Amaryllidaceae |
| *Crinum* L. | 6 (2) | Amaryllidaceae |
| *Cryptostephanus* Welw. ex Baker | 1 | Amaryllidaceae |
| *Cyrtanthus* Aiton | 2 (1) | Amaryllidaceae |
| *Habranthus* Herb. | (1) | Amaryllidaceae |
| *Hippeastrum* Herb. | (1) | Amaryllidaceae |
| *Hymenocallis* Salisb. | (1) | Amaryllidaceae |
| *Nerine* Herb. | (1) | Amaryllidaceae |
| *Nothoscordum* Kunth | (1) | Amaryllidaceae |
| *Pancratium* L. | 1 | Amaryllidaceae |
| *Scadoxus* Raf. | 1 | Amaryllidaceae |
| *Sprekelia* Heist. | (1) | Amaryllidaceae |
| *Zephyranthes* Herb. | (5) | Amaryllidaceae |
| f63. **Asparagaceae** |  |  |
| *Albuca* L. | 3 | Hyacinthaceae |
| *Anthericum* L. | 3 | Anthericaceae |
| *Asparagus* L. | 17 | Asparagaceae |
| *Bowiea* Harv. ex Hook. f. | 1 | Hyacinthaceae |
| *Chlorophytum* Ker Gawl. | 26 | Anthericaceae |
| *Dipcadi* Medik. | 3 | Hyacinthaceae |
| *Dracaena* Vand. ex L. | 7 | Dracaenaceae |
| *Drimia* Jacq. ex Willd. | 7 | Hyacinthaceae |
| *Drimiopsis* Lindl. et Paxton | 1 | Hyacinthaceae |
| *Eriospermum* Jacq. ex Willd. | 2 | Eriospermaceae |
| *Ledebouria* Roth | 5 | Hyacinthaceae |
| *Ornithogalum* L. | 3 | Hyacinthaceae |
| *Sansevieria* Thunb. | 26 (1) | Dracaenaceae |
| **Arecales** |  |  |
| f64. **Arecaceae** |  |  |
| *Borassus* L. | 1 | Palmae |
| *Cocos* L. | (1) | Palmae |
| *Elaeis* Jacq. | 1 | Palmae |
| *Hyphaene* Gaertn. | 3 | Palmae |
| *Phoenix* L. | 1 (1) | Palmae |
| *Raphia* P. Beauv. | 1 | Palmae |
| **Commelinales** |  |  |
| f65. **Commelinaceae** |  |  |
| *Aneilema* R. Br. | 28 | Commelinaceae |
| *Anthericopsis* Engl. | 1 | Commelinaceae |
| *Callisia* L. | (4) | Commelinaceae |
| *Coleotrype* C. B. Clarke | 1 | Commelinaceae |
| *Commelina* L. | 27 | Commelinaceae |
| *Cyanotis* D. Don | 9 (1) | Commelinaceae |
| *Dichorisandra* J. C. Mikan | (1) | Commelinaceae |
| *Floscopa* Lour. | 1 | Commelinaceae |
| *Murdannia* Royle | 3 | Commelinaceae |
| *Pollia* Thunb. | 1 | Commelinaceae |
| *Tradescantia* L. | (5) | Commelinaceae |
| f66. **Pontederiaceae** |  |  |
| *Eichhornia* Kunth | (1) | Pontederiaceae |
| *Heteranthera* Ruiz et Pav. | 1 | Pontederiaceae |
| *Monochoria* C. Presl | 1 | Pontederiaceae |
| **Zingiberales** |  |  |
| f67. **Musaceae** |  |  |
| *Ensete* Horan. | 1 | Musaceae |
| f68. **Cannaceae** |  |  |
| *Canna* L. | (1) | Musaceae |
| f69. **Costaceae** |  |  |
| *Costus* L. | 3 | Zingiberaceae |
| f70. **Zingiberaceae** |  |  |
| *Aframomum* K. Schum. | 6 | Zingiberaceae |
| *Renealmia* L. f. | 1 | Zingiberaceae |
| *Siphonochilus* J. M. Wood et Franks | 2 | Zingiberaceae |
| **Poales** |  |  |
| f71. **Typhaceae** |  |  |
| *Typha* L. | 2 | Typhaceae |
| f72. **Xyridaceae** |  |  |
| *Xyris* L. | 7 | Xyridaceae |
| f73. **Eriocaulaceae** |  |  |
| *Eriocaulon* L. | 7 | Eriocaulaceae |
| f74. **Juncaceae** |  |  |
| *Juncus* L. | 4 | Juncaceae |
| *Luzula* DC. | 3 | Juncaceae |
| f75. **Cyperaceae** |  |  |
| *Abildgaardia* Vahl | 2 | Cyperaceae |
| *Alinula* J. Raynal | 3 | Cyperaceae |
| *Ascolepis* Nees ex Steud. | 2 | Cyperaceae |
| *Bolboschoenus* (Asch.) Palla | 1 | Cyperaceae |
| *Bulbostylis* Kunth | 30 | Cyperaceae |
| *Carex* L. | 18 | Cyperaceae |
| *Cladium* P. Browne | 1 | Cyperaceae |
| *Coleochloa* Gilly | 1 | Cyperaceae |
| *Courtoisina* Soják | 2 | Cyperaceae |
| *Cyperus* L. | 94 | Cyperaceae |
| *Diplacrum* R. Br. | 1 | Cyperaceae |
| *Eleocharis* R. Br. | 8 | Cyperaceae |
| *Ficinia* Schrad. | 1 | Cyperaceae |
| *Fimbristylis* Vahl | 12 | Cyperaceae |
| *Fuirena* Rottb. | 13 | Cyperaceae |
| *Isolepis* R. Br. | 6 | Cyperaceae |
| *Kyllinga* Rottb. | 21 | Cyperaceae |
| *Kyllingiella* R. W. Haines et Lye | 2 | Cyperaceae |
| *Lipocarpha* R. Br. | 5 | Cyperaceae |
| *Oxycaryum* Nees | 1 | Cyperaceae |
| *Pycreus* P. Beauv. | 18 | Cyperaceae |
| *Queenslandiella* Domin | 1 | Cyperaceae |
| *Remirea* Aubl. | 1 | Cyperaceae |
| *Rhynchospora* Vahl | 2 | Cyperaceae |
| *Schoenoplectiella* Lye | 6 | Cyperaceae |
| *Schoenoplectus* (Rchb.) Palla | 3 | Cyperaceae |
| *Schoenoxiphium* Nees | 2 | Cyperaceae |
| *Scleria* P. J. Bergius | 16 | Cyperaceae |
| *Tetraria* P. Beauv. | 1 | Cyperaceae |
| f76. **Flagellariaceae** |  |  |
| *Flagellaria* L. | 1 | Flagellariaceae |
| f77. **Poaceae** |  |  |
| *Acrachne* Wight et Arn. ex Chiov. | 1 | Gramineae |
| *Acritochaete* Pilg. | 1 | Gramineae |
| *Acroceras* Stapf | 2 | Gramineae |
| *Agrostis* L. | 10 | Gramineae |
| *Aira* L. | 1 | Gramineae |
| *Alloteropsis* J. Presl | 4 | Gramineae |
| *Andropogon* L. | 13 | Gramineae |
| *Anthephora* Schreb. | 2 | Gramineae |
| *Anthoxanthum* L. | 1 | Gramineae |
| *Aristida* L. | 16 | Gramineae |
| *Arthraxon* P. Beauv. | 2 | Gramineae |
| *Arundinaria* Michx. | 1 | Gramineae |
| *Arundinella* Raddi | 1 | Gramineae |
| *Asthenatherum* Nevski | 1 | Gramineae |
| *Avena* L. | (2) | Gramineae |
| *Axonopus* P. Beauv. | 1 | Gramineae |
| *Bothriochloa* Kuntze | 3 | Gramineae |
| *Brachiaria* (Trin.) Griseb. | 31 | Gramineae |
| *Brachyachne* (Benth. et Hook. f.) Stapf | 1 | Gramineae |
| *Brachypodium* P. Beauv. | 1 | Gramineae |
| *Briza* L. | (1) | Gramineae |
| *Bromus* L. | 2 (2) | Gramineae |
| *Calamagrostis* Adans. | 2 | Gramineae |
| *Capillipedium* Stapf | 1 | Gramineae |
| *Cenchrus* L. | 6 | Gramineae |
| *Chloachne* Stapf | 1 | Gramineae |
| *Chloris* Sw. | 10 | Gramineae |
| *Chrysochloa* Swallen | 1 | Gramineae |
| *Chrysopogon* Trin. | 2 | Gramineae |
| *Cleistachne* Benth. | 1 | Gramineae |
| *Coelachne* R. Br. | 2 | Gramineae |
| *Coelachyrum* Hochst. et Nees | 1 | Gramineae |
| *Coelorachis* Brongn. | 1 | Gramineae |
| *Coelorachis* Brongn. | 1 | Gramineae |
| *Coix* L. | (1) | Gramineae |
| *Colpodium* Trin. | 2 | Gramineae |
| *Ctenium* Panz. | 2 | Gramineae |
| *Cymbopogon* Spreng. | 6 | Gramineae |
| *Cynodon* Rich. | 5 | Gramineae |
| *Cypholepis* Chiov. | 1 | Gramineae |
| *Cyrtococcum* Stapf | 1 | Gramineae |
| *Dactyloctenium* Willd. | 9 | Gramineae |
| *Daknopholis* Clayton | 1 | Gramineae |
| *Deschampsia* P. Beauv. | 3 | Gramineae |
| *Dichanthium* Willemet | 2 | Gramineae |
| *Digitaria* Haller | 23 | Gramineae |
| *Dignathia* Stapf | 2 | Gramineae |
| *Diheteropogon* (Hack.) Stapf | 1 | Gramineae |
| *Dinebra* Jacq. | 2 | Gramineae |
| *Diplachne* P. Beauv. | 2 | Gramineae |
| *Drake-brockmania* Stapf | 2 | Gramineae |
| *Echinochloa* P. Beauv. | 7 | Gramineae |
| *Ehrharta* Thunb. | 1 | Gramineae |
| *Eleusine* Gaertn. | 7 (1) | Gramineae |
| *Elionurus* Humb. et Bonpl. ex Willd. | 3 | Gramineae |
| *Enneapogon* Desv. ex P. Beauv. | 7 | Gramineae |
| *Entolasia* Stapf | 1 | Gramineae |
| *Eragrostiella* Bor | 1 | Gramineae |
| *Eragrostis* Wolf | 50 | Gramineae |
| *Eriochloa* Kunth | 5 | Gramineae |
| *Eriochrysis* P. Beauv. | 1 | Gramineae |
| *Eulalia* Kunth | 2 | Gramineae |
| *Eustachys* Desv. | 1 | Gramineae |
| *Exotheca* Andersson | 1 | Gramineae |
| *Festuca* L. | 7 (1) | Gramineae |
| *Gastridium* P. Beauv. | 1 | Gramineae |
| *Hackelochloa* Kuntze | 1 | Gramineae |
| *Halopyrum* Stapf | 1 | Gramineae |
| *Harpachne* A. Rich. | 2 | Gramineae |
| *Helictotrichon* Besser ex Schult. et Schult. f. | 5 | Gramineae |
| *Hemarthria* R. Br. | 1 | Gramineae |
| *Heteropogon* Pers. | 2 | Gramineae |
| *Holcolemma* Stapf et C. E. Hubb. | 1 | Gramineae |
| *Hylebates* Chippin. | 1 | Gramineae |
| *Hyparrhenia* Andersson ex E. Fourn. | 27 | Gramineae |
| *Hyperthelia* Clayton | 1 | Gramineae |
| *Imperata* Cyrillo | 1 | Gramineae |
| *Isachne* R. Br. | 1 | Gramineae |
| *Ischaemum* L. | 2 | Gramineae |
| *Koeleria* Pers. | 1 | Gramineae |
| *Leersia* Sol. et Swartz | 3 | Gramineae |
| *Leptaspis* R. Br. | 1 | Gramineae |
| *Leptocarydion* Hochst. ex Stapf | 1 | Gramineae |
| *Leptochloa* P. Beauv. | 5 | Gramineae |
| *Leptothrium* Kunth | 1 | Gramineae |
| *Lepturus* R. Br. | 2 | Gramineae |
| *Lintonia* Stapf | 2 | Gramineae |
| *Lolium* L. | 1 | Gramineae |
| *Loudetia* Hochst. ex Steud. | 5 | Gramineae |
| *Megastachya* P. Beauv. | 1 | Gramineae |
| *Melinis* P. Beauv. | 3 | Gramineae |
| *Microchloa* R. Br. | 2 | Gramineae |
| *Miscanthus* Andersson | 1 | Gramineae |
| *Olyra* L. | 1 | Gramineae |
| *Oplismenus* P. Beauv. | 4 | Gramineae |
| *Oropetium* Trin. | 3 | Gramineae |
| *Oryza* L. | 3 | Gramineae |
| *Panicum* L. | 34 (1) | Gramineae |
| *Paspalidium* Stapf | 4 | Gramineae |
| *Paspalum* L. | 5 | Gramineae |
| *Pennisetum* Rich. | 19 | Gramineae |
| *Pentaschistis* (Nees) Spach | 3 | Gramineae |
| *Perotis* Aiton | 2 | Gramineae |
| *Phalaris* L. | 1 (2) | Gramineae |
| *Phragmites* Adans. | 3 | Gramineae |
| *Poa* L. | 3 (1) | Gramineae |
| *Pogonarthria* Stapf | 1 | Gramineae |
| *Polypogon* Desf. | 1 | Gramineae |
| *Pseudechinolaena* Stapf | 1 | Gramineae |
| *Pseudobromus* K. Schum. | 2 | Gramineae |
| *Psilolemma* S. M. Phillips | 1 | Gramineae |
| *Rhynchelytrum* Nees | 4 | Gramineae |
| *Rhytachne* Desv. ex Ham. | 1 | Gramineae |
| *Rottboellia* L. f. | 1 | Gramineae |
| *Saccharum* L. | 1 | Gramineae |
| *Sacciolepis* Nash | 5 | Gramineae |
| *Schizachyrium* Nees | 5 | Gramineae |
| *Schmidtia* Steud. ex J. A. Schmidt | 1 | Gramineae |
| *Schoenefeldia* Kunth | 1 | Gramineae |
| *Sehima* Forssk. | 2 | Gramineae |
| *Setaria* P. Beauv. | 17 (1) | Gramineae |
| *Snowdenia* C. E. Hubbard | 2 | Gramineae |
| *Sorghastrum* Nash | 1 | Gramineae |
| *Sorghum* Moench | 3 | Gramineae |
| *Sporobolus* R. Br. | 39 | Gramineae |
| *Stenotaphrum* Trin. | 1 (1) | Gramineae |
| *Stipa* L. | 1 | Gramineae |
| *Stipagrostis* Nees | 2 | Gramineae |
| *Streblochaete* Hochst. ex A. Rich. | 1 | Gramineae |
| *Tetrachaete* Chiov. | 1 | Gramineae |
| *Tetrapogon* Desf. | 3 | Gramineae |
| *Themeda* Forssk. | 1 | Gramineae |
| *Trachypogon* Nees | 1 | Gramineae |
| *Tragus* Haller | 3 | Gramineae |
| *Tricholaena* Schrad. | 2 | Gramineae |
| *Trichoneura* Anderss. | 2 | Gramineae |
| *Tripogon* Roem. et Schult. | 5 | Gramineae |
| *Urochloa* P. Beauv. | 8 | Gramineae |
| *Vossia* Wall. et Griff. | 1 | Gramineae |
| *Vulpia* C. C. Gmel. | 1 (1) | Gramineae |
| **Ceratophyllales** |  |  |
| f78. **Ceratophyllaceae** |  |  |
| *Ceratophyllum* L. | 2 | Ceratophyllaceae |
| **Ranunculales** |  |  |
| f79. **Papaveraceae** |  |  |
| *Argemone* L. | (1) | Papaveraceae |
| *Corydalis* DC. | 1 | Fumariaceae |
| f80. **Menispermaceae** |  |  |
| *Chasmanthera* Hochst. | 1 | Menispermaceae |
| *Cissampelos* L. | 3 | Menispermaceae |
| *Cocculus* DC. | 2 | Menispermaceae |
| *Dioscoreophyllum* Engl. | 1 | Menispermaceae |
| *Jateorhiza* Miers | 1 | Menispermaceae |
| *Stephania* Lour. | 2 | Menispermaceae |
| *Tiliacora* Colebr. | 3 | Menispermaceae |
| *Tinospora* Miers | 3 | Menispermaceae |
| *Triclisia* Benth. | 1 | Menispermaceae |
| f81. **Berberidaceae** |  |  |
| *Berberis* L. | 1 | Berberidaceae |
| f82. **Ranunculaceae** |  |  |
| *Anemone* L. | 1 | Ranunculaceae |
| *Clematis* L. | 2 | Ranunculaceae |
| *Clematopsis* Bojer ex Hutch. | 1 | Ranunculaceae |
| *Delphinium* L. | 3 | Ranunculaceae |
| *Ranunculus* L. | 7 | Ranunculaceae |
| *Thalictrum* L. | 2 | Ranunculaceae |
| **Proteales** |  |  |
| f83. **Proteaceae** |  |  |
| *Faurea* Harv. | 4 | Proteaceae |
| *Grevillea* R. Br. | (1) | Proteaceae |
| *Protea* L. | 3 | Proteaceae |
| **Buxales** |  |  |
| f84. **Buxaceae** |  |  |
| *Notobuxus* Oliv. | 1 | Buxaceae |
| **Gunnerales** |  |  |
| f85. **Myrothamnaceae** |  |  |
| *Myrothamnus* Welw. | 1 | Myrothamnaceae |
| f86. **Gunneraceae** |  |  |
| *Gunnera* L. | 1 | Haloragaceae |
| **Dilleniales** |  |  |
| f87. **Dilleniaceae** |  |  |
| *Tetracera* L. | 2 | Dilleniaceae |
| **Saxifragales** |  |  |
| f88. **Hamamelidaceae** |  |  |
| *Trichocladus* Pers. | 1 | Hamamelidaceae |
| f89. **Crassulaceae** |  |  |
| *Aeonium* Webb et Berthel. | 1 | Crassulaceae |
| *Bryophyllum* Salisb. | 1 | Crassulaceae |
| *Cotyledon* Tourn. ex L. | 1 | Crassulaceae |
| *Crassula* L. | 10 | Crassulaceae |
| *Kalanchoe* Adans. | 18 | Crassulaceae |
| *Sedum* L. | 3 | Crassulaceae |
| *Umbilicus* DC. | 1 | Crassulaceae |
| f90. **Haloragaceae** |  |  |
| *Laurembergia* P. J. Bergius | 1 | Haloragaceae |
| *Myriophyllum* L. | 1 | Haloragaceae |
| **Vitales** |  |  |
| f91. **Vitaceae** |  |  |
| *Ampelocissus* Planch. | 2 | Vitaceae |
| *Cayratia* Juss. | 2 | Vitaceae |
| *Cissus* L. | 18 | Vitaceae |
| *Cyphostemma* (Planch.) Alston | 39 | Vitaceae |
| *Rhoicissus* Planch. | 2 | Vitaceae |
| **Zygophyllales** |  |  |
| f92. **Zygophyllaceae** |  |  |
| *Balanites* Delile | 5 | Balanitaceae |
| *Fagonia* L. | 3 | Zygophyllaceae |
| *Tribulus* L. | 6 | Zygophyllaceae |
| *Zygophyllum* L. | 1 | Zygophyllaceae |
| **Fabales** |  |  |
| f93. **Fabaceae** |  |  |
| *Abrus* Adans. | 4 | Leguminosae |
| *Acacia* Mill. | 44 (1) | Leguminosae |
| *Aeschynomene* L. | 10 | Leguminosae |
| *Afzelia* Sm. | 1 | Leguminosae |
| *Albizia* Durazz. | 13 | Leguminosae |
| *Alysicarpus* Neck. ex Desv. | 7 | Leguminosae |
| *Amphicarpaea* Elliott ex Nutt. (*Amphicarpa* Elliott ex Nutt. of FTEA) | 1 | Leguminosae |
| *Angylocalyx* Taub. | 1 | Leguminosae |
| *Antopetitia* A. Rich. | 1 | Leguminosae |
| *Argyrolobium* Eckl. et Zeyh. | 5 | Leguminosae |
| *Astragalus* L. | 1 | Leguminosae |
| *Baphia* Afzel. ex Lodd. | 1 | Leguminosae |
| *Bauhinia* L. | 3 | Leguminosae |
| *Bolusanthus* Harms | (1) | Leguminosae |
| *Brachystegia* Benth. | 1 | Leguminosae |
| *Cadia* Forssk. | 1 | Leguminosae |
| *Caesalpinia* L. | 6 (2) | Leguminosae |
| *Cajanus* DC. | 1 | Leguminosae |
| *Calliandra* Benth. | (2) | Leguminosae |
| *Calopogonium* Desv. | 1 | Leguminosae |
| *Calpurnia* E. Mey. | 4 (1) | Leguminosae |
| *Cassia* L. | 26 (4) | Leguminosae |
| *Castanospermum* A. Cunn. | (1) | Leguminosae |
| *Centrosema* Benth. | (1) | Leguminosae |
| *Ceratonia* L. | (1) | Leguminosae |
| *Clitoria* L. | 1 | Leguminosae |
| *Colutea* L. | 1 | Leguminosae |
| *Cordyla* Lour. | 1 | Leguminosae |
| *Craibia* Dunn | 4 | Leguminosae |
| *Crotalaria* L. | 93 | Leguminosae |
| *Cynometra* L. | 3 | Leguminosae |
| *Cytisus* L. | (1) | Leguminosae |
| *Dalbergia* L. f. | 8 (3) | Leguminosae |
| *Delonix* Raf. | 2 | Leguminosae |
| *Derris* Lour. | 1 | Leguminosae |
| *Desmodium* Desv. | 11 | Leguminosae |
| *Dialium* L. | 1 | Leguminosae |
| *Dichrostachys* (DC.) Wight et Arn. | 3 | Leguminosae |
| *Dicraeopetalum* Harms | 1 | Leguminosae |
| *Dolichos* L. | 6 | Leguminosae |
| *Dumasia* DC. | 1 | Leguminosae |
| *Entada* Adans. | 3 | Leguminosae |
| *Enterolobium* Mart. | (1) | Leguminosae |
| *Eriosema* (DC.) G. Don | 18 | Leguminosae |
| *Erythrina* L. | 5 | Leguminosae |
| *Erythrophleum* R. Br. | 1 | Leguminosae |
| *Flemingia* Roxb. ex W. T. Aiton | 1 | Leguminosae |
| *Galactia* P. Browne | 2 | Leguminosae |
| *Galega* L. | 2 | Leguminosae |
| *Gigasiphon* Drake | 1 | Leguminosae |
| *Gleditsia* L. | (1) | Leguminosae |
| *Glycine* Willd. | 1 | Leguminosae |
| *Haematoxylum* L. | (1) | Leguminosae |
| *Hymenaea* L. | (1) | Leguminosae |
| *Indigofera* L. | 70 | Leguminosae |
| *Julbernardia* Pellegr. | 1 | Leguminosae |
| *Kotschya* Endl. | 4 | Leguminosae |
| *Lablab* Adans. | 1 | Leguminosae |
| *Lathyrus* L. | 2 (4) | Leguminosae |
| *Lens* Mill. | (1) | Leguminosae |
| *Leucaena* Benth. | 1 | Leguminosae |
| *Lonchocarpus* Kunth | 2 | Leguminosae |
| *Lotononis* (DC.) Eckl. et Zeyh | 3 | Leguminosae |
| *Lotus* L. | 4 | Leguminosae |
| *Lupinus* L. | 1 (3) | Leguminosae |
| *Macrotyloma* (Wight et Arn.) Verdc. | 6 | Leguminosae |
| *Medicago* L. | 3 | Leguminosae |
| *Melilotus* (L.) Mill. | 3 | Leguminosae |
| *Millettia* Wight et Arn. | 6 | Leguminosae |
| *Mimosa* L. | 2 | Leguminosae |
| *Mucuna* Adans. | 4 | Leguminosae |
| *Mundulea* Benth. | 1 | Leguminosae |
| *Neorautanenia* Schinz | 1 | Leguminosae |
| *Neptunia* Lour. | 1 | Leguminosae |
| *Newtonia* Baill. | 4 | Leguminosae |
| *Ophrestia* H. M. L. Forbes | 2 | Leguminosae |
| *Ormocarpum* P. Beauv. | 6 | Leguminosae |
| *Paramacrolobium* J. Léonard | 1 | Leguminosae |
| *Parkia* R. Br. | 1 | Leguminosae |
| *Parkinsonia* L. | 2 (1) | Leguminosae |
| *Parochetus* Buch.-Ham. ex D. Don | 1 | Leguminosae |
| *Peltophorum* (Vogel) Benth. | (1) | Leguminosae |
| *Phaseolus* L. | (3) | Leguminosae |
| *Piliostigma* Hochst. | 1 | Leguminosae |
| *Pithecellobium* Mart. | (1) | Leguminosae |
| *Platycelyphium* Harms | 1 | Leguminosae |
| *Pseudarthria* Wight et Arn. | 2 | Leguminosae |
| *Pseudoeriosema* Hauman | 1 | Leguminosae |
| *Pseudovigna* (Harms) Verde | 1 | Leguminosae |
| *Psophocarpus* Neck. ex DC. | 1 | Leguminosae |
| *Psoralea* L. | 1 | Leguminosae |
| *Pterocarpus* Jacq. | (1) | Leguminosae |
| *Pterogyne* Tul. | (1) | Leguminosae |
| *Pterolobium* R. Br. ex Wight et Arn. | 1 | Leguminosae |
| *Pycnospora* R. Br. ex Wight et Arn. | 1 | Leguminosae |
| *Rhynchosia* Lour. | 28 | Leguminosae |
| *Rothia* Pers. | 1 | Leguminosae |
| *Scorodophloeus* Harms | 1 | Leguminosae |
| *Sesbania* Scop. | 10 | Leguminosae |
| *Sophora* L. | 2 (3) | Leguminosae |
| *Spartium* L. | (1) | Leguminosae |
| *Spathionema* Taub. | 1 | Leguminosae |
| *Sphenostylis* E. Mey. | 1 | Leguminosae |
| *Stylosanthes* Sw. | 2 | Leguminosae |
| *Tamarindus* L. | 1 | Leguminosae |
| *Tephrosia* Pers. | 27 (1) | Leguminosae |
| *Teramnus* P. Browne | 3 | Leguminosae |
| *Tetrapleura* Benth. | 1 | Leguminosae |
| *Tipuana* Benth. | (1) | Leguminosae |
| *Trachylobium* Hayne | 1 | Leguminosae |
| *Trifolium* L. | 16 | Leguminosae |
| *Tylosema* (Schweinf.) Torre et Hillc. | 3 | Leguminosae |
| *Vatovaea* Chiov. | 1 | Leguminosae |
| *Vicia* L. | 4 (6) | Leguminosae |
| *Vigna* Savi | 21 (1) | Leguminosae |
| *Virgilia* Lam. | (1) | Leguminosae |
| *Voandzeia* Thou. | (1) | Leguminosae |
| *Xeroderris* Roberty | 1 | Leguminosae |
| *Zornia* J. F. Gmel. | 6 | Leguminosae |
| f94. **Surianaceae** |  |  |
| *Suriana* L. | 1 | Surianaceae |
| f95. **Polygalaceae** |  |  |
| *Carpolobia* G. Don | 1 | Polygalaceae |
| *Polygala* L. | 30 | Polygalaceae |
| *Securidaca* L. | 2 | Polygalaceae |
| **Rosales** |  |  |
| f96. **Rosaceae** |  |  |
| *Alchemilla* L. | 13 | Rosaceae |
| *Cliffortia* L. | 1 | Rosaceae |
| *Fragaria* L. | 1 | Rosaceae |
| *Hagenia* J. F. Gmel. | 1 | Rosaceae |
| *Pygeum* Gaertn. | 1 | Rosaceae |
| *Rubus* L. | 10 | Rosaceae |
| f97. **Rhamnaceae** |  |  |
| *Berchemia* Neck. ex DC. | 1 | Rhamnaceae |
| *Colubrina* Rich. ex Brongn. | 1 | Rhamnaceae |
| *Gouania* Jacq. | 1 | Rhamnaceae |
| *Helinus* E. Meyer ex Endl. | 2 | Rhamnaceae |
| *Lasiodiscus* Hook. f. | 1 | Rhamnaceae |
| *Maesopsis* Engl. | 1 | Rhamnaceae |
| *Rhamnus* L. | 2 | Rhamnaceae |
| *Scutia* (Comm. ex DC.) Brongn. | 1 | Rhamnaceae |
| *Ventilago* Gaertn. | 1 | Rhamnaceae |
| *Ziziphus* Mill. | 6 | Rhamnaceae |
| f98. **Ulmaceae** |  |  |
| *Chaetacme* Rendle | 1 | Ulmaceae |
| f99. **Cannabaceae** |  |  |
| *Cannabis* L. | (1) | Cannabaceae |
| *Celtis* L. | 4 | Ulmaceae |
| *Trema* Lour. | 1 | Ulmaceae |
| f100. **Moraceae** |  |  |
| *Antiaris* Lesch. | 1 | Moraceae |
| *Dorstenia* L. | 15 | Moraceae |
| *Ficus* L. | 35 | Moraceae |
| *Maclura* Nutt. | 1 | Moraceae |
| *Milicia* Sim | 1 | Moraceae |
| *Morus* L. | 1 | Moraceae |
| *Sloetiopsis* Engl. | 1 | Moraceae |
| *Trilepisium* Thouars | 1 | Moraceae |
| f101. **Urticaceae** |  |  |
| *Australina* Gaudich. | 1 | Urticaceae |
| *Boehmeria* Jacq. | 1 | Urticaceae |
| *Didymodoxa* E. Mey. ex Wedd. | 1 | Urticaceae |
| *Droguetia* Gaudich. | 2 | Urticaceae |
| *Elatostema* J. R. Forst. et G. Forst. | 1 | Urticaceae |
| *Forsskaolea* L. | 1 | Urticaceae |
| *Girardinia* Gaudich. | 2 | Urticaceae |
| *Laportea* Gaudich. | 5 | Urticaceae |
| *Myrianthus* P. Beauv. | 1 | Cecropiaceae |
| *Obetia* Gaudich. | 1 | Urticaceae |
| *Parietaria* L. | 1 | Urticaceae |
| *Pilea* Lindl. | 5 | Urticaceae |
| *Pouzolzia* Gaudich. | 2 | Urticaceae |
| *Urera* Gaudich. | 3 | Urticaceae |
| **Fagales** |  |  |
| f102. **Casuarinaceae** |  |  |
| *Casuarina* Adans. | 1 (4) | Casuarinaceae |
| **Cucurbitales** |  |  |
| f103. **Cucurbitaceae** |  |  |
| *Cephalopentandra* Chiov. | 1 | Cucurbitaceae |
| *Citrullus* Schrad. | 2 | Cucurbitaceae |
| *Coccinia* Wight et Arn. | 10 | Cucurbitaceae |
| *Corallocarpus* Welw. ex Benth. et Hook. f. | 4 | Cucurbitaceae |
| *Ctenolepis* Hook. f. | 1 | Cucurbitaceae |
| *Cucumella* Chiov. | 2 | Cucurbitaceae |
| *Cucumis* L. | 10 | Cucurbitaceae |
| *Cyclantheropsis* Harms | 1 | Cucurbitaceae |
| *Dactyliandra* Hook. f. | 1 | Cucurbitaceae |
| *Diplocyclos* (Endl.) Post et O. Kuntze | 3 | Cucurbitaceae |
| *Eureiandra* Hook. f. | 2 | Cucurbitaceae |
| *Gerrardanthus* Harv. ex Benth. et Hook. | 2 | Cucurbitaceae |
| *Kedrostis* Medik. | 6 | Cucurbitaceae |
| *Lagenaria* Ser. | 2 (1) | Cucurbitaceae |
| *Luffa* Mill. | (1) | Cucurbitaceae |
| *Momordica* L. | 16 | Cucurbitaceae |
| *Mukia* Arn. | 1 | Cucurbitaceae |
| *Myrmecosicyos* C. Jeffrey | 1 | Cucurbitaceae |
| *Oreosyce* Hook. f. | 2 | Cucurbitaceae |
| *Peponium* Engl. | 2 | Cucurbitaceae |
| *Sicyos* L. | (1) | Cucurbitaceae |
| *Trochomeria* Hook. f. | 3 | Cucurbitaceae |
| *Zehneria* Endl. | 8 | Cucurbitaceae |
| f104. **Begoniaceae** |  |  |
| *Begonia* L. | 6 (2) | Begoniaceae |
| **Celastrales** |  |  |
| f105. **Celastraceae** |  |  |
| *Apodostigma* R. Wilczek | 1 | Celastraceae |
| *Catha* Forssk. | 1 | Celastraceae |
| *Elachyptera* A. C. Smith | 1 | Celastraceae |
| *Elaeodendron* Jacq. | 4 | Celastraceae |
| *Loeseneriella* A. C. Sm. | 2 | Celastraceae |
| *Maytenus* Molina | 12 | Celastraceae |
| *Mystroxylon* Eckl. et Zeyn. | 1 | Celastraceae |
| *Pleurostylia* Wight et Arn. | 1 | Celastraceae |
| *Pristimera* Miers | 4 | Celastraceae |
| *Salacia* L. | 7 | Celastraceae |
| *Simirestis* N. Hallé | 3 | Celastraceae |
| **Oxalidales** |  |  |
| f106. **Connaraceae** |  |  |
| *Agelaea* Sol. ex Planch. | 3 | Connaraceae |
| *Byrsocarpus* Schumach. | 2 | Connaraceae |
| *Connarus* L. | 1 | Connaraceae |
| *Ellipanthus* Hook. f. | 1 | Connaraceae |
| *Jaundea* Gilg | 1 | Connaraceae |
| *Santaloides* G. Schellenb. | 1 | Connaraceae |
| f107. **Oxalidaceae** |  |  |
| *Biophytum* DC. | 2 | Oxalidaceae |
| *Oxalis* L. | 4 (1) | Oxalidaceae |
| **Malpighiales** |  |  |
| f108. **Rhizophoraceae** |  |  |
| *Bruguiera* Lam. | 1 | Rhizophoraceae |
| *Cassipourea* Aubl. | 6 | Rhizophoraceae |
| *Ceriops* Arn. | 1 | Rhizophoraceae |
| *Rhizophora* L. | 1 | Rhizophoraceae |
| f109. **Erythroxylaceae** |  |  |
| *Erythroxylum* P. Browne | 3 | Erythroxylaceae |
| *Nectaropetalum* Engl. | 1 | Erythroxylaceae |
| f110. **Ochnaceae** |  |  |
| *Brackenridgea* A. Gray | 1 | Ochnaceae |
| *Gomphia* Schreb. | 5 | Ochnaceae |
| *Ochna* L. | 12 | Ochnaceae |
| f111. **Clusiaceae** |  |  |
| *Garcinia* L. | 3 | Guttiferae |
| f112. **Calophyllaceae** |  |  |
| *Calophyllum* L. | 1 | Guttiferae |
| f113. **Podostemaceae** |  |  |
| *Ledermanniella* Engl. | 1 | Podostemaceae |
| *Sphaerothylax* Bisch. ex Krauss | 1 | Podostemaceae |
| *Tristicha* Thouars | 1 | Podostemaceae |
| f114. **Hypericaceae** |  |  |
| *Harungana* Lamarck | 1 | Hyperiaceae |
| *Hypericum* L. | 11 | Hyperiaceae |
| *Psorospermum* Spach | 1 | Hyperiaceae |
| *Vismia* Vandelli | 1 | Hyperiaceae |
| f115. **Putranjivaceae** |  |  |
| *Drypetes* Vahl | 5 | Euphorbiaceae |
| f116. **Elatinaceae** |  |  |
| *Bergia* L. | 2 | Elatinaceae |
| f117. **Malpighiaceae** |  |  |
| *Acridocarpus* Guill. et Perr. | 4 | Malpighiaceae |
| *Caucanthus* Forsk. | 2 | Malpighiaceae |
| *Flabellaria* Cav. | 1 | Malpighiaceae |
| *Triaspis* Burch. | 3 | Malpighiaceae |
| *Tristellateia* Thouars | 1 | Malpighiaceae |
| f118. **Dichapetalaceae** |  |  |
| *Dichapetalum* Thouars | 8 | Dichapetalaceae |
| *Tapura* Aubl. | 1 | Dichapetalaceae |
| f119. **Chrysobalanaceae** |  |  |
| *Hirtella* L. | 1 | Rosaceae |
| *Parinari* Aubl. | 1 | Rosaceae |
| f120. **Achariaceae** |  |  |
| *Dasylepis* Oliver | 1 | Flacourtiaceae |
| *Grandidiera* Jaub. | 1 | Flacourtiaceae |
| *Lindackeria* C. Presl | 1 | Flacourtiaceae |
| *Rawsonia* Harv. et Sond. | 1 | Flacourtiaceae |
| *Xylotheca* Hochst. | 1 (1) | Flacourtiaceae |
| f121. **Violaceae** |  |  |
| *Hybanthus* Jacq. | 2 | Violaceae |
| *Rinorea* Aubl. | 8 | Violaceae |
| *Viola* L. | 3 | Violaceae |
| f122. **Passifloraceae** |  |  |
| *Adenia* Forssk. | 18 | Passifloraceae |
| *Basananthe* Peyr. | 4 | Passifloraceae |
| *Loewia* Urb. | 2 | Turneraceae |
| *Passiflora* L. | (6) | Passifloraceae |
| *Schlechterina* Harms | 1 | Passifloraceae |
| *Streptopetalum* Hochst. | 2 | Turneraceae |
| *Wormskioldia* Thoss. | 3 | Turneraceae |
| f123. **Salicaceae** |  |  |
| *Bivinia* Tul. | 1 | Flacourtiaceae |
| *Casearia* Jacq. | 2 | Flacourtiaceae |
| *Dovyalis* E. Mey. ex Arn. | 4 (1) | Flacourtiaceae |
| *Flacourtia* Comm. ex L'Hér. | 1 | Flacourtiaceae |
| *Homalium* Jacq. | 1 | Flacourtiaceae |
| *Ludia* Comm. ex Juss. | 1 | Flacourtiaceae |
| *Oncoba* Forssk. | 2 | Flacourtiaceae |
| *Populus* L. | 1 (2) | Salicaceae |
| *Salix* L. | 1 | Salicaceae |
| *Scolopia* Schreb. | 4 | Flacourtiaceae |
| *Trimeria* Harv. | 1 | Flacourtiaceae |
| f124. **Peraceae** |  |  |
| *Clutia* L. | 2 | Euphorbiaceae |
| f125. **Euphorbiaceae** |  |  |
| *Acalypha* L. | 19 | Euphorbiaceae |
| *Alchornea* Sw. | 3 | Euphorbiaceae |
| *Aleurites* J. R. Forst. et G. Forst. | (1) | Euphorbiaceae |
| *Argomuellera* Pax | 1 | Euphorbiaceae |
| *Caperonia* A. St.-Hil. | 2 | Euphorbiaceae |
| *Cavacoa* J. Léonard | 1 | Euphorbiaceae |
| *Cephalocroton* Hochst. | 1 | Euphorbiaceae |
| *Chrozophora* A. Juss. | 1 | Euphorbiaceae |
| *Codiaeum* A. Juss. | (1) | Euphorbiaceae |
| *Croton* L. | 14 (1) | Euphorbiaceae |
| *Dalechampia* L. | 11 | Euphorbiaceae |
| *Euphorbia* L. | 95 (10) | Euphorbiaceae |
| *Excoecaria* L. | 2 | Euphorbiaceae |
| *Givotia* Griff. | 1 | Euphorbiaceae |
| *Homalanthus* A. Juss. | (1) | Euphorbiaceae |
| *Hura* L. | 1 | Euphorbiaceae |
| *Jatropha* L. | 14 (5) | Euphorbiaceae |
| *Joannesia* Vell. | (1) | Euphorbiaceae |
| *Macaranga* Thouars | 4 | Euphorbiaceae |
| *Mallotus* Lour. | 1 | Euphorbiaceae |
| *Manihot* Mill. | (2) | Euphorbiaceae |
| *Micrococca* Benth. | 3 | Euphorbiaceae |
| *Mildbraedia* Pax | 2 | Euphorbiaceae |
| *Monadenium* Pax | 14 | Euphorbiaceae |
| *Neoboutonia* Müll. Arg. | 2 | Euphorbiaceae |
| *Neoholstia* Rauschert | 1 | Euphorbiaceae |
| *Pedilanthus* Neck. ex Poit. | (1) | Euphorbiaceae |
| *Ricinodendron* Müll. Arg. | 1 | Euphorbiaceae |
| *Ricinus* L. | 1 | Euphorbiaceae |
| *Sapium* Jacq. | 1 | Euphorbiaceae |
| *Spirostachys* Sond. | 2 | Euphorbiaceae |
| *Suregada* Roxb. ex Rottler | 2 | Euphorbiaceae |
| *Synadenium* Boiss. | 5 | Euphorbiaceae |
| *Tragia* Plum. ex L. | 13 | Euphorbiaceae |
| *Tragiella* Pax et K. Hoffm. | 1 | Euphorbiaceae |
| *Vernicia* Lour. | (2) | Euphorbiaceae |
| f126. **Linaceae** |  |  |
| *Hugonia* L. | 1 | Linaceae |
| *Linum* L. | 3 | Linaceae |
| f127. **Picrodendraceae** |  |  |
| *Aristogeitonia* Prain | 1 | Euphorbiaceae |
| *Oldfieldia* Benth. et Hook. f. | 1 | Euphorbiaceae |
| f128. **Phyllanthaceae** |  |  |
| *Andrachne* L. | 1 | Euphorbiaceae |
| *Antidesma* L. | 3 | Euphorbiaceae |
| *Bischofia* Blume | (1) | Euphorbiaceae |
| *Breynia* J. R. Forst. et G. Forst. | 1 | Euphorbiaceae |
| *Bridelia* Willd. | 5 | Euphorbiaceae |
| *Cleistanthus* Hook. f. ex Planch. | 3 | Euphorbiaceae |
| *Cyathogyne* Müll. Arg. | 1 | Euphorbiaceae |
| *Flueggea* Willd. | 1 | Euphorbiaceae |
| *Heywoodia* Sim | 1 | Euphorbiaceae |
| *Hymenocardia* Wall. ex Lindl. | 1 | Euphorbiaceae |
| *Margaritaria* L. f. | 1 | Euphorbiaceae |
| *Meineckia* Baill. | 2 | Euphorbiaceae |
| *Phyllanthus* L. | 28 | Euphorbiaceae |
| *Savia* Willd. | 1 | Euphorbiaceae |
| *Thecacoris* A. Juss. | 1 | Euphorbiaceae |
| *Zimmermannia* Pax | 1 | Euphorbiaceae |
| **Geraniales** |  |  |
| f129. **Geraniaceae** |  |  |
| *Geranium* L. | 6 | Geraniaceae |
| *Monsonia* L. | 4 | Geraniaceae |
| *Pelargonium* L'Hér. ex Aiton | 4 (1) | Geraniaceae |
| f130. **Francoaceae** |  |  |
| *Bersama* Fresen. | 1 | Melianthaceae |
| **Myrtales** |  |  |
| f131. **Combretaceae** |  |  |
| *Combretum* Loefl. | 22 | Combretaceae |
| *Lumnitzera* Willd. | 1 | Combretaceae |
| *Pteleopsis* Engl. | 2 | Combretaceae |
| *Quisqualis* L. | 1 | Combretaceae |
| *Terminalia* L. | 11 | Combretaceae |
| f132. **Lythraceae** |  |  |
| *Ammannia* L. | 7 | Lythraceae |
| *Cuphea* P. Browne | (1) | Lythraceae |
| *Lawsonia* L. | 1 | Lythraceae |
| *Lythrum* L. | 1 | Lythraceae |
| *Nesaea* Comm. ex Kunth | 9 | Lythraceae |
| *Pemphis* J. R. Forst. et G. Forst. | 1 | Lythraceae |
| *Rotala* L. | 7 | Lythraceae |
| *Sonneratia* L. f. | 1 | Sonneratiaceae |
| *Woodfordia* Salisb. | 1 | Lythraceae |
| f133. **Onagraceae** |  |  |
| *Epilobium* L. | 3 | Onagraceae |
| *Fuchsia* L. | (1) | Onagraceae |
| *Jussiaea* L. | 6 | Onagraceae |
| f134. **Myrtaceae** |  |  |
| *Acca* O. Berg | (1) | Myrtaceae |
| *Agonis* (DC.) Sweet | (1) | Myrtaceae |
| *Angophora* Cav. | (1) | Myrtaceae |
| *Callistemon* R. Br. | (7) | Myrtaceae |
| *Calothamnus* Labill. | (1) | Myrtaceae |
| *Eucalyptus* L'Hér. | (99) | Myrtaceae |
| *Eugenia* L. | 6 (2) | Myrtaceae |
| *Leptospermum* J. R. Forst. et G. Forst. | (3) | Myrtaceae |
| *Lophostemon* Schott | (1) | Myrtaceae |
| *Melaleuca* L. | (11) | Myrtaceae |
| *Metrosideros* Banks ex Gaertn. | (1) | Myrtaceae |
| *Psidium* L. | (2) | Myrtaceae |
| *Syncarpia* Ten. | (1) | Myrtaceae |
| *Syzygium* Gaertn. | 4 (2) | Myrtaceae |
| f135. **Melastomataceae** |  |  |
| *Antherotoma* Hook. f. | 1 | Melastomataceae |
| *Dissotis* Benth. | 6 | Melastomataceae |
| *Memecylon* L. | 7 | Melastomataceae |
| *Tristemma* Juss. | 1 | Melastomataceae |
| f136. **Penaeaceae** |  |  |
| *Olinia* Thunb. | 1 | Oliniaceae |
| **Crossosomatales** |  |  |
| f137. **Aphloiaceae** |  |  |
| *Aphloia* (DC.) Benn. | 1 | Flacourtiaceae |
| **Sapindales** |  |  |
| f138. **Kirkiaceae** |  |  |
| *Kirkia* Oliv. | 1 | Simaroubaceae |
| f139. **Burseraceae** |  |  |
| *Boswellia* Roxb. ex Colebr. | 3 | Burseraceae |
| *Commiphora* Jacq. | 51 | Burseraceae |
| f140. **Anacardiaceae** |  |  |
| *Anacardium* (L.) Rottboell | (1) | Anacardiaceae |
| *Lannea* A. Rich. | 11 | Anacardiaceae |
| *Mangifera* L. | (1) | Anacardiaceae |
| *Ozoroa* Delile | 2 | Anacardiaceae |
| *Pistacia* L. | 1 | Anacardiaceae |
| *Pseudospondias* Engl. | 1 | Anacardiaceae |
| *Rhus* Tourn. ex L. | 5 | Anacardiaceae |
| *Schinus* L. | (1) | Anacardiaceae |
| *Sclerocarya* Hochst. | 2 | Anacardiaceae |
| *Sorindeia* Thou. | 1 | Anacardiaceae |
| f141. **Sapindaceae** |  |  |
| *Allophylus* L. | 6 | Sapindaceae |
| *Aporrhiza* Radlk. | 1 | Sapindaceae |
| *Blighia* K. D. Koenig | 1 | Sapindaceae |
| *Camptolepis* Radlk. | 1 | Sapindaceae |
| *Cardiospermum* L. | 3 | Sapindaceae |
| *Chytranthus* Hook. f. | 2 | Sapindaceae |
| *Deinbollia* Schumacher et Thonn. | 2 | Sapindaceae |
| *Dodonaea* Mill. | 1 | Sapindaceae |
| *Filicium* Thwaites | 1 | Sapindaceae |
| *Glenniea* Hook. f. | 1 | Sapindaceae |
| *Haplocoelopsis* F. G. Davies | 1 | Sapindaceae |
| *Haplocoelum* Radlk. | 2 | Sapindaceae |
| *Lecaniodiscus* Planch. ex Benth. | 1 | Sapindaceae |
| *Lepisanthes* Blume | 1 | Sapindaceae |
| *Macphersonia* Blume | 1 | Sapindaceae |
| *Majidea* J. Kirk ex Oliv. | 2 | Sapindaceae |
| *Pancovia* Willd. | 2 | Sapindaceae |
| *Pappea* Eckl. et Zeyh. | 1 | Sapindaceae |
| *Paullinia* L. | 1 | Sapindaceae |
| *Sapindus* L. | 1 | Sapindaceae |
| *Stadmania* Lamarck | 1 | Sapindaceae |
| *Zanha* Hiern | 2 | Sapindaceae |
| f142. **Rutaceae** |  |  |
| *Bottegoa* Chiov. | 1 | Ptaeroxylaceae |
| *Calodendrum* Thunb. | 1 | Rutaceae |
| *Casimiroa* La Llave et Lex. | (1) | Rutaceae |
| *Choisya* Kunth | (1) | Rutaceae |
| *Citrus* L. | (8) | Rutaceae |
| *Clausena* Burm. f. | 1 | Rutaceae |
| *Diphasia* Pierre | 1 | Rutaceae |
| *Diphasiopsis* Mendon?a | 1 | Rutaceae |
| *Fagaropsis* Mildbraed | 2 | Rutaceae |
| *Flindersia* R. Br. | (1) | Rutaceae |
| *Murraya* J. Koenig ex L. | (2) | Rutaceae |
| *Ruta* L. | (1) | Rutaceae |
| *Teclea* Delile | 6 | Rutaceae |
| *Toddalia* A. Juss. | 1 | Rutaceae |
| *Toddaliopsis* Engl. | 1 | Rutaceae |
| *Vepris* Comm. ex A. Juss. | 6 | Rutaceae |
| *Zanthoxylum* L. | 7 | Rutaceae |
| f143. **Simaroubaceae** |  |  |
| *Brucea* J. F. Mill. | 2 | Simaroubaceae |
| *Quassia* L. | 1 | Simaroubaceae |
| f144. **Meliaceae** |  |  |
| *Azadirachta* A. Juss. | (1) | Meliaceae |
| *Cedrela* P. Browne | (1) | Meliaceae |
| *Ekebergia* Sparrm. | 1 | Meliaceae |
| *Entandrophragma* C. DC. | 1 | Meliaceae |
| *Lepidotrichilia* (Harms) J. F. Leroy | 1 | Meliaceae |
| *Lovoa* Harms | 1 | Meliaceae |
| *Melia* L. | 2 | Meliaceae |
| *Pseudobersama* Verde | 1 | Meliaceae |
| *Toona* (Endl.) M. Roem. | (2) | Meliaceae |
| *Trichilia* P. Browne | 2 | Meliaceae |
| *Turraea* L. | 12 | Meliaceae |
| *Xylocarpus* J. Koenig | 2 | Meliaceae |
| **Malvales** |  |  |
| f145. **Muntingiaceae** |  |  |
| *Muntingia* L. | (1) | Muntingiaceae |
| f146. **Malvaceae** |  |  |
| *Abelmoschus* Medik. | 1 | Malvaceae |
| *Abutilon* Mill. | 17 | Malvaceae |
| *Adansonia* L. | 1 | Bombacaceae |
| *Anoda* Cav. | (1) | Malvaceae |
| *Bombacopsis* Pittier | (1) | Bombacaceae |
| *Bombax* L. | 1 | Bombacaceae |
| *Brachychiton* Schott et Endl. | (2) | Sterculiaceae |
| *Byttneria* Loefl. | 1 | Sterculiaceae |
| *Carpodiptera* Griseb. | 1 | Tiliaceae |
| *Chorisia* Kunth | (1) | Bombacaceae |
| *Christiana* DC. | 1 | Tiliaceae |
| *Cienfuegosia* Cav. | 1 | Malvaceae |
| *Cola* Schott et Endl. | 6 (1) | Sterculiaceae |
| *Corchorus* L. | 11 | Tiliaceae |
| *Dombeya* Cav. | 7 | Sterculiaceae |
| *Fremontodendron* Coville | (1) | Sterculiaceae |
| *Gossypioides* Skovsted | 1 | Malvaceae |
| *Gossypium* L. | 4 | Malvaceae |
| *Grewia* L. | 33 | Tiliaceae |
| *Guazuma* Mill. | (1) | Sterculiaceae |
| *Harmsia* K. Schum. | 1 | Sterculiaceae |
| *Heritiera* Aiton | 1 | Sterculiaceae |
| *Hermannia* L. | 14 | Sterculiaceae |
| *Hibiscus* L. | 37 (4) | Malvaceae |
| *Kleinhovia* L. | (1) | Sterculiaceae |
| *Kosteletzkya* C. Presl | 3 | Malvaceae |
| *Lagunaria* (DC.) Rchb. | (1) | Malvaceae |
| *Leptonychia* Turcz. | 1 | Sterculiaceae |
| *Malva* L. | 2 | Malvaceae |
| *Malvastrum* A. Gray | 1 | Malvaceae |
| *Malvaviscus* Fabr. | (2) | Malvaceae |
| *Melhania* Forssk. | 11 | Sterculiaceae |
| *Melochia* L. | 2 | Sterculiaceae |
| *Nesogordonia* Baill. | 1 | Sterculiaceae |
| *Pavonia* Cav. | 21 | Malvaceae |
| *Pterospermum* Schreb. | (1) | Sterculiaceae |
| *Roifia* Verdc. | 1 | Malvaceae |
| *Senra* Cav. | 1 | Malvaceae |
| *Sida* L. | 13 | Malvaceae |
| *Sparrmannia* L. f. | 1 | Tiliaceae |
| *Sterculia* L. | 5 (1) | Sterculiaceae |
| *Theobroma* L. | (1) | Sterculiaceae |
| *Thespesia* Sol. ex Corrêa | 3 | Malvaceae |
| *Triumfetta* L. | 11 | Tiliaceae |
| *Urena* L. | 1 | Malvaceae |
| *Waltheria* L. | 1 | Sterculiaceae |
| *Wissadula* Medik. | 1 | Malvaceae |
| f147. **Thymelaeaceae** |  |  |
| *Dicranolepis* Planch. | 1 | Thymelaeaceae |
| *Gnidia* L. | 10 | Thymelaeaceae |
| *Peddiea* Harv. | 1 | Thymelaeaceae |
| *Struthiola* L. | 1 | Thymelaeaceae |
| *Synaptolepis* Oliv. | 1 | Thymelaeaceae |
| f148. **Bixaceae** |  |  |
| *Bixa* L. | 1 | Bixaceae |
| **Brassicales** |  |  |
| f149. **Moringaceae** |  |  |
| *Moringa* Adans. | 7 | Moringaceae |
| f150. **Caricaceae** |  |  |
| *Carica* L. | (1) | Caricaceae |
| *Cylicomorpha* Urb. | 1 | Caricaceae |
| f151. **Salvadoraceae** |  |  |
| *Azima* Lam. | 1 | Salvadoraceae |
| *Dobera* Juss. | 2 | Salvadoraceae |
| *Salvadora* Garcin ex L. | 1 | Salvadoraceae |
| f152. **Resedaceae** |  |  |
| *Caylusea* A. St.-Hil. | 2 | Resedaceae |
| *Reseda* L. | 2 | Resedaceae |
| f153. **Capparaceae** |  |  |
| *Boscia* Lam. | 7 | Capparidaceae |
| *Cadaba* Forssk. | 12 | Capparidaceae |
| *Capparis* Tourn. ex L. | 6 | Capparidaceae |
| *Cladostemon* A. Braun et Vatke | 1 | Capparidaceae |
| *Crateva* L. | 1 | Capparidaceae |
| *Maerua* Forssk. | 20 | Capparidaceae |
| *Ritchiea* R. Br. | 2 | Capparidaceae |
| *Thilachium* Lour. | 2 | Capparidaceae |
| f154. **Cleomaceae** |  |  |
| *Cleome* L. | 14 | Capparidaceae |
| *Gynandropsis* DC. | 1 | Capparidaceae |
| f155. **Brassicaceae** |  |  |
| *Arabidopsis* (DC.) Heynh. | 1 | Cruciferae |
| *Arabis* L. | 1 | Cruciferae |
| *Barbarea* R. Br. | 1 | Cruciferae |
| *Brassica* L. | 5 | Cruciferae |
| *Camelina* Crantz | 1 | Cruciferae |
| *Capsella* Medik. | (1) | Cruciferae |
| *Cardamine* L. | 4 (1) | Cruciferae |
| *Coronopus* Zinn | (1) | Cruciferae |
| *Crambe* L. | 2 | Cruciferae |
| *Diceratella* Boiss. | 3 | Cruciferae |
| *Erucastrum* Webb et Berth. ex C. Presl | 1 | Cruciferae |
| *Farsetia* Turra | 7 | Cruciferae |
| *Lepidium* L. | 2 (2) | Cruciferae |
| *Matthiola* R. Br. | 1 | Cruciferae |
| *Oreophyton* O. E. Schulz | 1 | Cruciferae |
| *Raphanus* L. | 2 | Cruciferae |
| *Rorippa* Scop. | 4 (1) | Cruciferae |
| *Sisymbrium* L. | 3 | Cruciferae |
| *Subularia* L. | 1 | Cruciferae |
| *Thlaspi* L. | 1 | Cruciferae |
| *Turritis* L. | 1 | Cruciferae |
| **Santalales** |  |  |
| f156. **Olacaceae** |  |  |
| *Strombosia* Blume | 1 | Olacaceae |
| *Ximenia* L. | 2 | Olacaceae |
| f157. **Opiliaceae** |  |  |
| *Opilia* Roxb. | 2 | Opiliaceae |
| *Rhopalopilia* Pierre | 1 | Opiliaceae |
| f158. **Balanophoraceae** |  |  |
| *Sarcophyte* Sparrm. | 1 | Balanophoraceae |
| *Thonningia* Vahl | 1 | Balanophoraceae |
| f159. **Santalaceae** |  |  |
| *Arceuthobium* M. Bieb. | 1 | Viscaceae |
| *Korthalsella* Tiegh. | 1 | Viscaceae |
| *Osyridicarpos* A. DC. | 1 | Santalaceae |
| *Osyris* L. | 1 | Santalaceae |
| *Thesium* L. | 10 | Santalaceae |
| *Viscum* L. | 9 | Santalaceae |
| f160. **Loranthaceae** |  |  |
| *Agelanthus* Tiegh. | 15 | Loranthaceae |
| *Emelianthe* Danser | 1 | Loranthaceae |
| *Englerina* Tiegh. | 5 | Loranthaceae |
| *Erianthemum* Tiegh. | 5 | Loranthaceae |
| *Globimetula* Tiegh. | 1 | Loranthaceae |
| *Helixanthera* Lour. | 2 | Loranthaceae |
| *Oliverella* Tiegh. | 1 | Loranthaceae |
| *Oncella* Tiegh. | 2 | Loranthaceae |
| *Oncocalyx* Tiegh. | 6 | Loranthaceae |
| *Phragmanthera* Tiegh. | 3 | Loranthaceae |
| *Plicosepalus* Tiegh. | 4 | Loranthaceae |
| *Spragueanella* Balle | 1 | Loranthaceae |
| *Tapinanthus* (Blume) Rchb. | 3 | Loranthaceae |
| *Taxillus* Tiegh. | 1 | Loranthaceae |
| **Caryophyllales** |  |  |
| f161. **Tamaricaceae** |  |  |
| *Tamarix* L. | 2 | Tamariaceae |
| f162. **Plumbaginaceae** |  |  |
| *Ceratostigma* Bunge | 1 | Plumbaginaceae |
| *Limonium* Mill. | 1 (3) | Plumbaginaceae |
| *Plumbago* L. | 4 | Plumbaginaceae |
| f163. **Polygonaceae** |  |  |
| *Antigonon* Endl. | (1) | Polygonaceae |
| *Emex* Neck. ex Campd. | (2) | Polygonaceae |
| *Fagopyrum* Mill. | (1) | Polygonaceae |
| *Harpagocarpus* Hutch. et Dandy | 1 | Polygonaceae |
| *Oxygonum* Burch. ex Campd. | 6 | Polygonaceae |
| *Polygonum* L. | 7 (4) | Polygonaceae |
| *Rumex* L. | 4 (2) | Polygonaceae |
| f164. **Droseraceae** |  |  |
| *Drosera* L. | 2 | Droseraceae |
| f165. **Ancistrocladaceae** |  |  |
| *Ancistrocladus* Wall. | 1 | Ancistrocladaceae |
| f166. **Caryophyllaceae** |  |  |
| *Agrostemma* L. | (1) | Caryophyllaceae |
| *Cerastium* L. | 3 | Caryophyllaceae |
| *Corrigiola* L. | 1 | Caryophyllaceae |
| *Drymaria* Willd. ex Schult. | 1 | Caryophyllaceae |
| *Krauseola* Pax et K. Hoffm. | 1 | Caryophyllaceae |
| *Pollichia* Ait. | 1 | Caryophyllaceae |
| *Polycarpaea* Lam. | 4 | Caryophyllaceae |
| *Sagina* L. | 2 | Caryophyllaceae |
| *Scleranthus* L. | (1) | Caryophyllaceae |
| *Silene* L. | 3 | Caryophyllaceae |
| *Spergula* L. | 1 | Caryophyllaceae |
| *Stellaria* L. | 3 | Caryophyllaceae |
| *Uebelinia* Hochst. | 1 | Caryophyllaceae |
| *Vaccaria* Wolf | (1) | Caryophyllaceae |
| f167. **Amaranthaceae** |  |  |
| *Achyranthes* L. | 1 | Amaranthaceae |
| *Achyropsis* (Moq.) Hook. f. | 1 | Amaranthaceae |
| *Aerva* Forssk. | 3 | Amaranthaceae |
| *Allmaniopsis* Suess. | 1 | Amaranthaceae |
| *Alternanthera* Forssk. | 1 (2) | Amaranthaceae |
| *Amaranthus* L. | 9 (1) | Amaranthaceae |
| *Arthrocnemum* Moq. | 1 | Chenopodiaceae |
| *Atriplex* L. | 1 (2) | Chenopodiaceae |
| *Celosia* L. | 6 | Chenopodiaceae |
| *Centemopsis* Schinz | 3 | Chenopodiaceae |
| *Centrostachys* Wall. | 1 | Chenopodiaceae |
| *Chenopodium* L. | 6 (3) | Chenopodiaceae |
| *Cyathula* Blume | 7 | Amaranthaceae |
| *Dasysphaera* Volkens ex Gilg | 1 | Amaranthaceae |
| *Digera* Forssk. | 1 | Amaranthaceae |
| *Gomphrena* L. | (2) | Amaranthaceae |
| *Guilleminea* Kunth | (1) | Amaranthaceae |
| *Hermbstaedtia* Rchb. | 1 | Amaranthaceae |
| *Iresine* P. Browne | (1) | Amaranthaceae |
| *Lopriorea* Schinz | 1 | Amaranthaceae |
| *Nothosaerva* Wight | 1 | Amaranthaceae |
| *Pandiaka* (Moq.) Hook. f. | 1 | Amaranthaceae |
| *Psilotrichum* Blume | 8 | Amaranthaceae |
| *Pupalia* Juss. | 2 | Amaranthaceae |
| *Salsola* L. | 1 | Chenopodiaceae |
| *Sericocomopsis* Schinz | 2 | Amaranthaceae |
| *Sericostachys* Gilg et Lopr. | 1 | Amaranthaceae |
| *Suaeda* Forssk. ex J. F. Gmel. | 1 | Amaranthaceae |
| *Volkensinia* Schinz | 1 | Amaranthaceae |
| f168. **Limeaceae** |  |  |
| *Limeum* L. | 2 | Aizoaceae |
| f169. **Lophiocarpaceae** |  |  |
| *Corbichonia* Scop. | 1 | Aizoaceae |
| f170. **Gisekiaceae** |  |  |
| *Gisekia* L. | 2 | Aizoaceae |
| f171. **Aizoaceae** |  |  |
| *Aizoon* L. | 1 | Aizoaceae |
| *Delosperma* N. E. Br. | 2 | Aizoaceae |
| *Sesuvium* L. | 2 | Aizoaceae |
| *Tetragonia* L. | (2) | Aizoaceae |
| *Trianthema* L. | 3 | Aizoaceae |
| *Tribulocarpus* S. Moore | 1 | Aizoaceae |
| *Zaleya* Burm. f. | 1 | Aizoaceae |
| f172. **Phytolaccaceae** |  |  |
| *Phytolacca* L. | 1 (1) | Phytolaccaceae |
| f173. **Petiveriaceae** |  |  |
| *Hilleria* Vell. | 1 | Phytolaccaceae |
| f174. **Nyctaginaceae** |  |  |
| *Boerhavia* L. | 6 | Nyctaginaceae |
| *Commicarpus* Standl. | 5 | Nyctaginaceae |
| *Mirabilis* L. | (1) | Nyctaginaceae |
| *Pisonia* L. | 2 | Nyctaginaceae |
| f175. **Molluginaceae** |  |  |
| *Glinus* L. | 3 | Aizoaceae |
| *Hypertelis* E. Mey. ex Fenzl | 1 | Aizoaceae |
| *Mollugo* L. | 2 | Aizoaceae |
| f176. **Montiaceae** |  |  |
| *Montia* L. | 1 | Portulacaceae |
| f177. **Didiereaceae** |  |  |
| *Calyptrotheca* Gilg | 2 | Portulacaceae |
| *Portulacaria* Jacq. | 1 | Portulacaceae |
| f178. **Basellaceae** |  |  |
| *Basella* L. | 2 | Basellaceae |
| f179. **Talinaceae** |  |  |
| *Talinum* Adans. | 4 (1) | Portulacaceae |
| f180. **Portulacaceae** |  |  |
| *Portulaca* L. | 18 | Portulacaceae |
| f181. **Cactaceae** |  |  |
| *Opuntia* Mill. | (4) | Cactaceae |
| *Rhipsalis* Gaertn. | 1 | Cactaceae |
| **Cornales** |  |  |
| f182. **Cornaceae** |  |  |
| *Afrocrania* (Harms) Hutch. | 1 | Cornaceae |
| *Alangium* Lam. | 2 | Alangiaceae |
| *Dendrobenthamia* Hutch. | (1) | Cornaceae |
| **Ericales** |  |  |
| f183. **Balsaminaceae** |  |  |
| *Impatiens* L. | 21 | Balsaminaceae |
| f184. **Lecythidaceae** |  |  |
| *Barringtonia* J. R. Forst. et G. Forst. | 1 | Lecythidaceae |
| f185. **Sapotaceae** |  |  |
| *Afrosersalisia* A. Chev. | 2 | Sapotaceae |
| *Aningeria* Aubrév. et Pellegr. | 2 | Sapotaceae |
| *Bequaertiodendron* de Wild. | 2 | Sapotaceae |
| *Chrysophyllum* L. | 3 | Sapotaceae |
| *Inhambanella* Dubard | 1 | Sapotaceae |
| *Malacantha* Pierre | 1 | Sapotaceae |
| *Manilkara* Adans. | 5 | Sapotaceae |
| *Mimusops* L. | 6 | Sapotaceae |
| *Pachystela* Pierre | 3 | Sapotaceae |
| *Sideroxylon* L. | 1 | Sapotaceae |
| *Vitellariopsis* Baill. ex Dubard | 1 | Sapotaceae |
| f186. **Ebenaceae** |  |  |
| *Diospyros* L. | 17 | Ebenaceae |
| *Euclea* Murray | 3 | Ebenaceae |
| f187. **Primulaceae** |  |  |
| *Anagallis* L. | 7 | Primulaceae |
| *Ardisiandra* Hook. f. | 2 | Primulaceae |
| *Asterolinon* Hoffmanns. et Link | 1 | Primulaceae |
| *Embelia* Burm. f. | 2 | Primulaceae |
| *Lysimachia* L. | 3 | Primulaceae |
| *Maesa* Forssk. | 1 | Myrsinaceae |
| *Myrsine* L. | 1 | Myrsinaceae |
| *Rapanea* Aubl. | 1 | Myrsinaceae |
| *Samolus* L. | 1 | Primulaceae |
| f188. **Ericaceae** |  |  |
| *Agarista* D. Don ex G. Don | 1 | Ericaceae |
| *Erica* L. | 7 | Ericaceae |
| **Icacinales** |  |  |
| f189. **Icacinaceae** |  |  |
| *Iodes* Blume | 1 | Icacinaceae |
| *Pyrenacantha* Hook. ex Wight | 2 | Icacinaceae |
| **Metteniusales** |  |  |
| f190. **Metteniusaceae** |  |  |
| *Apodytes* E. Mey. ex Arn. | 1 | Icacinaceae |
| **Gentianales** |  |  |
| f191. **Rubiaceae** |  |  |
| *Agathisanthemum* Klotzsch | 1 | Rubiaceae |
| *Anthospermum* L. | 3 | Rubiaceae |
| *Borreria* G. Mey. | 1 | Rubiaceae |
| *Breonadia* Ridsdale | 1 | Rubiaceae |
| *Burchellia* R. Br. | (1) | Rubiaceae |
| *Calycosiphonia* (Pierre) Lebrun | 1 | Rubiaceae |
| *Canthium* Lam. | 12 | Rubiaceae |
| *Carphalea* Juss. | 1 | Rubiaceae |
| *Catunaregam* Wolf | 2 | Rubiaceae |
| *Chassalia* Comm. ex Poir. | 5 | Rubiaceae |
| *Chazaliella* E. M. A. Petit et Verdc. | 1 | Rubiaceae |
| *Cinchona* L. | (1) | Rubiaceae |
| *Cladoceras* Bremek. | 1 | Rubiaceae |
| *Coffea* L. | 6 | Rubiaceae |
| *Conostomium* (Stapf) Cufod. | 2 | Rubiaceae |
| *Craterispermum* Benth. | 1 | Rubiaceae |
| *Cremaspora* Benth. | 1 | Rubiaceae |
| *Crossopteryx* Fenzl | 1 | Rubiaceae |
| *Dibrachionostylus* Bremek. | 1 | Rubiaceae |
| *Didymosalpinx* Keay | 1 | Rubiaceae |
| *Diodia* L. | 2 | Rubiaceae |
| *Fadogia* Schweinf. | 1 | Rubiaceae |
| *Feretia* Delile | 1 | Rubiaceae |
| *Galiniera* Delile | 1 | Rubiaceae |
| *Galium* L. | 11 | Rubiaceae |
| *Gardenia* J. Ellis | 5 | Rubiaceae |
| *Geophila* D. Don | 2 | Rubiaceae |
| *Guettarda* L. | 1 | Rubiaceae |
| *Fleroya* Y. F. Deng (*Hallea* J.-F. Leroy of FTEA) | 1 | Rubiaceae |
| *Heinsenia* K. Schum. | 1 | Rubiaceae |
| *Heinsia* DC. | 2 | Rubiaceae |
| *Hymenodictyon* Wall. | 2 | Rubiaceae |
| *Ixora* L. | 2 | Rubiaceae |
| *Keetia* E. P. Phillips | 3 | Rubiaceae |
| *Kohautia* Cham. et Schltdl. | 7 | Rubiaceae |
| *Kraussia* Harv. | 2 | Rubiaceae |
| *Lagynias* E. Mey. | 1 | Rubiaceae |
| *Lamprothamnus* Hiern | 1 | Rubiaceae |
| *Lasianthus* Jack | 1 | Rubiaceae |
| *Leptactina* Hook. f. | 1 | Rubiaceae |
| *Luculia* Sweet | (1) | Rubiaceae |
| *Meyna* Roxb. ex Link | 1 | Rubiaceae |
| *Mitracarpus* Zucc. | 1 | Rubiaceae |
| *Mitriostigma* Hochst. | 1 | Rubiaceae |
| *Multidentia* A. Gilli | 2 | Rubiaceae |
| *Mussaenda* L. | 3 (2) | Rubiaceae |
| *Oldenlandia* L. | 19 | Rubiaceae |
| *Otomeria* Benth. | 2 | Rubiaceae |
| *Oxyanthus* DC. | 5 | Rubiaceae |
| *Pachystigma* Hochst. | 3 | Rubiaceae |
| *Paederia* L. | 1 | Rubiaceae |
| *Paraknoxia* Bremek. | 1 | Rubiaceae |
| *Parapentas* Bremek. | 1 | Rubiaceae |
| *Pauridiantha* Hook. f. | 1 | Rubiaceae |
| *Pavetta* L. | 19 | Rubiaceae |
| *Pentanisia* Harv. | 4 | Rubiaceae |
| *Pentanopsis* Rendle | 1 | Rubiaceae |
| *Pentas* Benth. | 11 | Rubiaceae |
| *Pentodon* Hochst. | 1 | Rubiaceae |
| *Polysphaeria* Hook. f. | 4 | Rubiaceae |
| *Portlandia* P. Browne | (1) | Rubiaceae |
| *Pseudomussaenda* Wernham | 1 | Rubiaceae |
| *Psychotria* L. | 22 | Rubiaceae |
| *Psydrax* Gaertn. | 9 | Rubiaceae |
| *Pyrostria* Comm. ex Juss. | 3 | Rubiaceae |
| *Richardia* L. | 1 | Rubiaceae |
| *Rothmannia* Thunb. | 6 | Rubiaceae |
| *Rubia* L. | 1 | Rubiaceae |
| *Rutidea* DC. | 3 | Rubiaceae |
| *Rytigynia* Blume | 12 | Rubiaceae |
| *Sarcocephalus* Afzel. ex R. Br. | 1 | Rubiaceae |
| *Spermacoce* L. | 12 | Rubiaceae |
| *Tarenna* Gaertn. | 9 | Rubiaceae |
| *Tennantia* B. Verdcourt | 1 | Rubiaceae |
| *Triainolepis* Hook. f. | 1 | Rubiaceae |
| *Tricalysia* A. Rich. ex DC. | 6 | Rubiaceae |
| *Uncaria* Schreb. | 1 | Rubiaceae |
| *Vangueria* Juss. | 5 | Rubiaceae |
| f192. **Gentianaceae** |  |  |
| *Anthocleista* Afzel. ex R. Br. | 2 | Loganiaceae |
| *Canscora* Lam. | 1 | Gentianaceae |
| *Chironia* L. | 1 | Gentianaceae |
| *Enicostema* Blume | 1 | Gentianaceae |
| *Exacum* L. | 1 | Gentianaceae |
| *Sebaea* Sol. ex R. Br. | 8 | Gentianaceae |
| *Swertia* L. | 15 | Gentianaceae |
| f193. **Loganiaceae** |  |  |
| *Strychnos* L. | 9 | Loganiaceae |
| f194. **Gelsemiaceae** |  |  |
| *Mostuea* Didr. | 3 | Loganiaceae |
| f195. **Apocynaceae** |  |  |
| *Acokanthera* G. Don | 2 | Apocynaceae |
| *Adenium* Roem. et Schult. | 1 | Apocynaceae |
| *Alafia* Thouars | 2 | Apocynaceae |
| *Allamanda* L. | (3) | Apocynaceae |
| *Alstonia* R. Br. | (1) | Apocynaceae |
| *Ancylobothrys* Pierre | 2 | Apocynaceae |
| *Araujia* Brot. | (1) | Apocynaceae |
| *Asclepias* L. | 1 (1) | Apocynaceae |
| *Aspidoglossum* E. Mey. | 3 | Apocynaceae |
| *Baissea* A. DC. | 2 | Apocynaceae |
| *Baseonema* Schlechter et Rendle | 1 | Apocynaceae |
| *Beaumontia* Wall. | (1) | Apocynaceae |
| *Blyttia* Arn. | 1 | Apocynaceae |
| *Brachystelma* R. Br. | 5 | Apocynaceae |
| *Buckollia* H. J. T. Venter et R. L. Verhoeven | 1 | Apocynaceae |
| *Calotropis* R. Br. | 2 | Apocynaceae |
| *Caralluma* R. Br. | 8 | Apocynaceae |
| *Carissa* L. | 3 | Apocynaceae |
| *Carvalhoa* K. Schum. | 1 | Apocynaceae |
| *Catharanthus* G. Don | (1) | Apocynaceae |
| *Ceropegia* L. | 31 (2) | Apocynaceae |
| *Chlorocyathus* Oliver | 1 | Apocynaceae |
| *Conomitra* Fenzl | 1 | Apocynaceae |
| *Cryptolepis* R. Br. | 5 | Apocynaceae |
| *Cryptostegia* R. Br. | (1) | Apocynaceae |
| *Cynanchum* L. | 11 | Apocynaceae |
| *Desmidorchis* Ehrenb. | 3 | Apocynaceae |
| *Dictyophleba* Pierre | 1 | Apocynaceae |
| *Diplostigma* K. Schum. | 1 | Apocynaceae |
| *Echidnopsis* Hook. f. | 11 | Apocynaceae |
| *Edithcolea* N. E. Br. | 1 | Apocynaceae |
| *Fockea* Endl. | 1 | Apocynaceae |
| *Funtumia* Stapf | 1 | Apocynaceae |
| *Glossonema* Decne. | 2 | Apocynaceae |
| *Gomphocarpus* R. Br. | 7 | Apocynaceae |
| *Holarrhena* R. Br. | 1 | Apocynaceae |
| *Huernia* R. Br. | 7 | Apocynaceae |
| *Hunteria* Roxb. | 2 | Apocynaceae |
| *Kanahia* R. Br. | 1 | Apocynaceae |
| *Landolphia* P. Beauv. | 4 | Apocynaceae |
| *Leptadenia* R. Br. | 1 | Apocynaceae |
| *Mandevilla* Lindl. | (2) | Apocynaceae |
| *Margaretta* Oliv. | 1 | Apocynaceae |
| *Marsdenia* R. Br. | 10 | Apocynaceae |
| *Mascarenhasia* A. DC. | 1 | Apocynaceae |
| *Mondia* Skeels | 2 | Apocynaceae |
| *Monolluma* D. C. H. Plowes | 1 | Apocynaceae |
| *Oncinotis* Benth. | 1 | Apocynaceae |
| *Orbea* Haw. | 12 | Apocynaceae |
| *Oxystelma* R. Br. | 1 | Apocynaceae |
| *Pachycarpus* E. Mey. | 6 | Apocynaceae |
| *Parquetina* Baill. | 1 | Apocynaceae |
| *Pentarrhinum* E. Mey. | 5 | Apocynaceae |
| *Pentatropis* R. Br. ex Wight et Arn. | 1 | Apocynaceae |
| *Pergularia* L. | 1 | Apocynaceae |
| *Periploca* L. | 1 | Apocynaceae |
| *Pleiocarpa* Benth. | 2 | Apocynaceae |
| *Pleioceras* Baill. | 1 | Apocynaceae |
| *Pleurostelma* Baillon. | 1 | Apocynaceae |
| *Plumeria* L. | (1) | Apocynaceae |
| *Raphionacme* Harv. | 6 | Apocynaceae |
| *Rauvolfia* L. | 3 | Apocynaceae |
| *Rhytidocaulon* P. R. O. Bally | 1 | Apocynaceae |
| *Saba* (Pichon) Pichon | 1 | Apocynaceae |
| *Sacleuxia* Baill. | 2 | Apocynaceae |
| *Schizostephanus* Hochst. ex Benth. et Hook. f. | 1 | Apocynaceae |
| *Schizozygia* Baill. | 1 | Apocynaceae |
| *Schlechterella* K. Schum. | 2 | Apocynaceae |
| *Secamone* R. Br. | 8 | Apocynaceae |
| *Stapelia* L. | (1) | Apocynaceae |
| *Stathmostelma* K. Schum. | 5 | Apocynaceae |
| *Stephanotis* Thouars | (1) | Apocynaceae |
| *Strophanthus* DC. | 5 | Apocynaceae |
| *Tabernaemontana* L. | 4 | Apocynaceae |
| *Tacazzea* Decne. | 2 | Apocynaceae |
| *Telosma* Coville | 1 | Apocynaceae |
| *Thevetia* L. | (1) | Apocynaceae |
| *Trachelospermum* Lem. | (1) | Apocynaceae |
| *Tylophora* R. Br. | 11 | Apocynaceae |
| *Vinca* L. | (1) | Apocynaceae |
| *Voacanga* Thouars | 2 | Apocynaceae |
| *Wrightia* R. Br. | 1 | Apocynaceae |
| *Xysmalobium* R. Br. | 3 | Apocynaceae |
| **Boraginales** |  |  |
| f196. **Boraginaceae** |  |  |
| *Argusia* Boehm. | 1 | Boraginaceae |
| *Bourreria* P. Browne | 4 | Boraginaceae |
| *Coldenia* L. | 1 | Boraginaceae |
| *Cordia* L. | 16 | Boraginaceae |
| *Cynoglossum* L. | 6 (1) | Boraginaceae |
| *Cystostemon* Balf. f. | 2 | Boraginaceae |
| *Echiochilon* Desf. | 1 | Boraginaceae |
| *Echium* L. | 1 | Boraginaceae |
| *Ehretia* L. | 5 | Boraginaceae |
| *Heliotropium* L. | 16 | Boraginaceae |
| *Lithospermum* L. | 1 | Boraginaceae |
| *Myosotis* L. | 3 | Boraginaceae |
| *Trichodesma* R. Br. | 7 | Boraginaceae |
| **Vahliales** |  |  |
| f197. **Vahliaceae** |  |  |
| *Vahlia* Thunb. | 3 | Vahliaceae |
| **Solanales** |  |  |
| f198. **Convolvulaceae** |  |  |
| *Astripomoea* A. Meeuse | 7 | Convolvulaceae |
| *Bonamia* Thouars | 1 | Convolvulaceae |
| *Cladostigma* Radlk. | 1 | Convolvulaceae |
| *Convolvulus* L. | 8 | Convolvulaceae |
| *Cressa* L. | 1 | Convolvulaceae |
| *Cuscuta* L. | 7 (1) | Convolvulaceae |
| *Dichondra* J. R. Forst. et G. Forst. | 1 | Convolvulaceae |
| *Evolvulus* L. | 2 | Convolvulaceae |
| *Falkia* Thunb. | 1 | Convolvulaceae |
| *Hewittia* Wight et Arn. | 1 | Convolvulaceae |
| *Hildebrandtia* Vatke | 4 | Convolvulaceae |
| *Ipomoea* L. | 57 (1) | Convolvulaceae |
| *Jacquemontia* Choisy | 3 | Convolvulaceae |
| *Lepistemon* Blume | 1 | Convolvulaceae |
| *Lepistemonopsis* Dammer | 1 | Convolvulaceae |
| *Merremia* Dennst. ex Endl. | 14 | Convolvulaceae |
| *Operculina* Silva Manso | 1 | Convolvulaceae |
| *Seddera* Hochst. | 3 | Convolvulaceae |
| *Stictocardia* Hallier f. | 3 | Convolvulaceae |
| *Turbina* Raf. | 1 | Convolvulaceae |
| f199. **Solanaceae** |  |  |
| *Browallia* L. | (1) | Solanaceae |
| *Brugmansia* Pers. | (1) | Solanaceae |
| *Brunfelsia* L. | (1) | Solanaceae |
| *Capsicum* L. | (2) | Solanaceae |
| *Cestrum* L. | (5) | Solanaceae |
| *Datura* L. | (3) | Solanaceae |
| *Discopodium* Hochst. | 2 | Solanaceae |
| *Iochroma* Benth. | (2) | Solanaceae |
| *Lycium* L. | 1 | Solanaceae |
| *Nicandra* Adans. | 1 | Solanaceae |
| *Nicotiana* L. | (4) | Solanaceae |
| *Physalis* L. | (3) | Solanaceae |
| *Schizanthus* Ruiz et Pav. | (1) | Solanaceae |
| *Solandra* Sw. | (1) | Solanaceae |
| *Solanum* L. | 41 (17) | Solanaceae |
| *Streptosolen* Miers | (1) | Solanaceae |
| *Withania* Pauquy | 1 | Solanaceae |
| F200. **Montiniaceae** |  |  |
| *Grevea* Baill. | 1 | Montiniaceae |
| **Lamiales** |  |  |
| f201. **Oleaceae** |  |  |
| *Dekindtia* Gilg | 1 | Oleaceae |
| *Jasminum* L. | 11 | Oleaceae |
| *Linociera* Sw. | 1 | Oleaceae |
| *Olea* L. | 4 | Oleaceae |
| *Schrebera* Roxb. | 1 | Oleaceae |
| f202. **Gesneriaceae** |  |  |
| *Saintpaulia* Wendl. | 1 | Gesneriaceae |
| *Streptocarpus* Lindl. | 7 | Gesneriaceae |
| f203. **Plantaginaceae** |  |  |
| *Bacopa* Aubl. | 2 | Scrophularlaceae |
| *Callitriche* L. | 4 | Callitrichaceae |
| *Dopatrium* Buch.-Ham. ex Benth. | 2 | Scrophularlaceae |
| *Misopates* Raf. | 1 | Scrophularlaceae |
| *Nanorrhinum* Betsche | 1 | Scrophularlaceae |
| *Plantago* L. | 4 (1) | Plantaginaceae |
| *Scoparia* L. | 1 | Scrophularlaceae |
| *Sibthorpia* L. | 1 | Scrophularlaceae |
| *Stemodia* L. | 1 | Scrophularlaceae |
| *Veronica* L. | 6 | Scrophularlaceae |
| f204. **Scrophulariaceae** |  |  |
| *Anticharis* Endl. | 2 | Scrophularlaceae |
| *Aptosimum* Burchell | 1 | Scrophularlaceae |
| *Buddleja* L. | 2 | Loganiaceae |
| *Diclis* Benth. | 2 | Scrophularlaceae |
| *Hebenstretia* L. | 1 | Scrophularlaceae |
| *Limosella* L. | 5 | Scrophularlaceae |
| *Selago* L. | 1 | Scrophularlaceae |
| *Verbascum* L. | 4 (1) | Scrophularlaceae |
| f205. **Stilbaceae** |  |  |
| *Halleria* L. | 1 | Scrophularlaceae |
| *Nuxia* Comm. ex Lam. | 3 | Loganiaceae |
| f206. **Linderniaceae** |  |  |
| *Artanema* D. Don | 1 | Scrophularlaceae |
| *Craterostigma* Hochst. | 7 | Scrophularlaceae |
| *Lindernia* All. | 16 | Scrophularlaceae |
| *Stemodiopsis* Engl. | 2 | Scrophularlaceae |
| *Torenia* L. | 3 | Scrophularlaceae |
| f207. **Pedaliaceae** |  |  |
| *Ceratotheca* Endl. | 2 | Pedaliaceae |
| *Josephinia* Vent. | 1 | Pedaliaceae |
| *Pedalium* Royen ex L. | 1 | Pedaliaceae |
| *Pterodiscus* Hook. | 2 | Pedaliaceae |
| *Sesamothamnus* Welw. | 2 | Pedaliaceae |
| *Sesamum* L. | 5 | Pedaliaceae |
| f208. **Acanthaceae** |  |  |
| *Acanthopale* C. B. Clarke | 2 | Acanthaceae |
| *Acanthus* L. | 3 | Acanthaceae |
| *Anisotes* Nees | 5 | Acanthaceae |
| *Aphelandra* R. Br. | (1) | Acanthaceae |
| *Asystasia* Blume | 14 | Acanthaceae |
| *Avicennia* L. | 1 | Verbenaceae |
| *Barleria* L. | 44 | Acanthaceae |
| *Blepharis* Juss. | 12 | Acanthaceae |
| *Brachystephanus* Nees | 1 | Acanthaceae |
| *Brillantaisia* P. Beauv. | 5 | Acanthaceae |
| *Cephalophis* Vollesen | 1 | Acanthaceae |
| *Chlamydacanthus* Lindau | 1 | Acanthaceae |
| *Crabbea* Harv. | 1 | Acanthaceae |
| *Crossandra* Salisb. | 11 | Acanthaceae |
| *Dicliptera* Juss. | 14 | Acanthaceae |
| *Duosperma* Dayton | 6 | Acanthaceae |
| *Dyschoriste* Nees | 7 | Acanthaceae |
| *Ecbolium* Kurz | 5 | Acanthaceae |
| *Elytraria* Michx. | 2 | Acanthaceae |
| *Eranthemum* L. | (1) | Acanthaceae |
| *Eremomastax* Lindau | 1 | Acanthaceae |
| *Graptophyllum* Nees | (1) | Acanthaceae |
| *Hygrophila* R. Br. | 6 | Acanthaceae |
| *Hypoestes* Sol. ex R. Br. | 4 | Acanthaceae |
| *Isoglossa* Oerst. | 8 | Acanthaceae |
| *Justicia* L. | 54 (2) | Acanthaceae |
| *Lankesteria* Lindl. | 1 | Acanthaceae |
| *Lepidagathis* Willd. | 6 | Acanthaceae |
| *Mackaya* Harv. | (1) | Acanthaceae |
| *Megalochlamys* Lindau | 6 | Acanthaceae |
| *Mendoncia* Vell. ex Vand. | 1 | Acanthaceae |
| *Mimulopsis* Schweinf. | 3 | Acanthaceae |
| *Monothecium* Hochst. | 2 | Acanthaceae |
| *Nelsonia* R. Br. | 1 | Acanthaceae |
| *Neuracanthus* Nees | 6 | Acanthaceae |
| *Phaulopsis* Willd. | 3 | Acanthaceae |
| *Pseuderanthemum* Radlk. | 2 | Acanthaceae |
| *Rhinacanthus* Nees | 5 | Acanthaceae |
| *Ruellia* L. | 7 | Acanthaceae |
| *Ruspolia* Lindau | 1 | Acanthaceae |
| *Ruttya* Harv. | 1 | Acanthaceae |
| *Sanchezia* Ruiz et Pav. | (1) | Acanthaceae |
| *Satanocrater* Schweinf. | 2 | Acanthaceae |
| *Sclerochiton* Harv. | 2 | Acanthaceae |
| *Thunbergia* Retz. | 19 (3) | Acanthaceae |
| *Trichaulax* Vollesen | 1 | Acanthaceae |
| *Whitfieldia* Hook. | 2 | Acanthaceae |
| f209. **Bignoniaceae** |  |  |
| *Adenocalymma* Mart. ex Meisn. | (1) | Bignoniaceae |
| *Anemopaegma* Mart. ex Meisn. | (1) | Bignoniaceae |
| *Clytostoma* Miers ex Bureau | (1) | Bignoniaceae |
| *Crescentia* L. | (1) | Bignoniaceae |
| *Distictis* Mart. ex Meisn. | (1) | Bignoniaceae |
| *Eccremocarpus* Ruiz et Pav. | (1) | Bignoniaceae |
| *Fernandoa* Welw. ex Seem. | 1 | Bignoniaceae |
| *Jacaranda* Juss. | (1) | Bignoniaceae |
| *Kigelia* DC. | 1 | Bignoniaceae |
| *Macfadyena* A. DC. | (1) | Bignoniaceae |
| *Mansoa* DC. | (1) | Bignoniaceae |
| *Markhamia* Seem. ex Baill. | 2 | Bignoniaceae |
| *Millingtonia* L. f. | (1) | Bignoniaceae |
| *Pandorea* Spach | (2) | Bignoniaceae |
| *Parmentiera* DC. | (1) | Bignoniaceae |
| *Pithecoctenium* Mart. ex Meisn. | (1) | Bignoniaceae |
| *Podranea* Sprague | (1) | Bignoniaceae |
| *Pyrostegia* C. Presl | (1) | Bignoniaceae |
| *Spathodea* P. Beauv. | 1 | Bignoniaceae |
| *Stereospermum* Cham. | 1 | Bignoniaceae |
| *Tabebuia* Gomes ex DC. | (5) | Bignoniaceae |
| *Tecoma* Juss. | (2) | Bignoniaceae |
| *Tecomanthe* Baill. | (1) | Bignoniaceae |
| *Tourrettia* Foug. | (1) | Bignoniaceae |
| f210. **Lentibulariaceae** |  |  |
| *Genlisea* A. St.-Hil. | 1 | Lentibulariaceae |
| *Utricularia* L. | 10 | Lentibulariaceae |
| f2111. **Verbenaceae** |  |  |
| *Chascanum* E. Mey. | 8 | Verbenaceae |
| *Citharexylum* L. | (1) | Verbenaceae |
| *Duranta* L. | (1) | Verbenaceae |
| *Lantana* L. | 3 (3) | Verbenaceae |
| *Lippia* L. | 7 | Verbenaceae |
| *Petrea* L. | (1) | Verbenaceae |
| *Phyla* Lour. | (1) | Verbenaceae |
| *Priva* Adans. | 3 | Verbenaceae |
| *Stachytarpheta* Vahl | 1 | Verbenaceae |
| *Verbena* L. | (5) | Verbenaceae |
| f212. **Lamiaceae** |  |  |
| *Achyrospermum* Blume | 3 | Lamiaceae |
| *Aeollanthus* Mart. ex Spreng. | 5 | Lamiaceae |
| *Ajuga* L. | 1 | Lamiaceae |
| *Basilicum* Moench | 1 | Lamiaceae |
| *Caryopteris* Bunge | (1) | Lamiaceae |
| *Clerodendrum* L. | 24 (1) | Verbenaceae |
| *Clinopodium* L. | 5 | Lamiaceae |
| *Congea* Roxb. | (1) | Verbenaceae |
| *Endostemon* N. E. Br. | 8 | Lamiaceae |
| *Fuerstia* T. C. E. Fries | 1 | Lamiaceae |
| *Gmelina* L. | (2) | Verbenaceae |
| *Haumaniastrum* P. A. Duvign. et Plancke | 3 | Lamiaceae |
| *Holmskioldia* Retz. | (1) | Verbenaceae |
| *Hoslundia* Vahl | 1 | Lamiaceae |
| *Hyptis* Jacq. | 3 | Lamiaceae |
| *Isodon* (Benth.) Kudo | 1 | Lamiaceae |
| *Karomia* Dop | 1 | Verbenaceae |
| *Lavandula* L. | (3) | Lamiaceae |
| *Leonotis* R. Br. | 2 | Lamiaceae |
| *Leucas* R. Br. | 21 | Lamiaceae |
| *Marrubium* L. | (1) | Lamiaceae |
| *Mentha* L. | 2 | Lamiaceae |
| *Micromeria* Benth. | 1 | Lamiaceae |
| *Moluccella* L. | (1) | Lamiaceae |
| *Nepeta* L. | 1 | Lamiaceae |
| *Ocimum* L. | 19 (1) | Lamiaceae |
| *Orthosiphon* Benth. | 5 | Lamiaceae |
| *Oxera* Labill. | (1) | Verbenaceae |
| *Phlomis* L. | (1) | Lamiaceae |
| *Platostoma* P. Beauv. | 4 | Lamiaceae |
| *Plectranthus* L'Hér. | 54 | Lamiaceae |
| *Premna* L. | 11 | Verbenaceae |
| *Pycnostachys* Hook. | 9 (1) | Lamiaceae |
| *Salvia* L. | 2 (8) | Lamiaceae |
| *Scutellaria* L. | 2 | Lamiaceae |
| *Stachys* L. | 4 | Lamiaceae |
| *Tectona* L. f. | (1) | Verbenaceae |
| *Tetradenia* Benth. | 1 | Lamiaceae |
| *Tinnea* Kotschy ex Hook. f. | 1 | Lamiaceae |
| *Vitex* L. | 10 | Verbenaceae |
| f213. **Phrymaceae** |  |  |
| *Glossostigma* Wight et Arn. | 1 | Scrophularlaceae |
| *Mimulus* L. | 1 | Scrophularlaceae |
| f214. **Orobanchaceae** |  |  |
| *Alectra* Thunb. | 8 | Scrophularlaceae |
| *Asepalum* Marais | 1 | Cyclocheilaceae |
| *Bartsia* L. | 3 | Scrophularlaceae |
| *Buchnera* L. | 5 | Scrophularlaceae |
| *Buttonia* MacKen ex Benth. | 1 | Scrophularlaceae |
| *Cistanche* Hoffmanns. et Link | 1 | Orobanchaceae |
| *Cycniopsis* Engl. | 1 | Scrophularlaceae |
| *Cycnium* E. Mey. ex Benth. | 12 | Scrophularlaceae |
| *Ghikaea* Volkens et Schweinf. | 1 | Scrophularlaceae |
| *Harveya* Hook. | 3 | Scrophularlaceae |
| *Hedbergia* Molau | 1 | Scrophularlaceae |
| *Lindenbergia* Lehm. | 1 | Scrophularlaceae |
| *Micrargeria* Benth. | 1 | Scrophularlaceae |
| *Orobanche* L. | 2 (1) | Orobanchaceae |
| *Pseudosopubia* Engl. | 2 | Scrophularlaceae |
| *Rhamphicarpa* Penth. | 1 | Scrophularlaceae |
| *Sopubia* Buch.-Ham. ex D. Don | 5 | Scrophularlaceae |
| *Striga* Lour. | 8 | Scrophularlaceae |
| **Aquifoliales** |  |  |
| f215. **Aquifoliaceae** |  |  |
| *Ilex* L. | 1 | Aquifoliaceae |
| **Asterales** |  |  |
| f216. **Campanulaceae** |  |  |
| *Campanula* L. | 2 | Campanulaceae |
| *Canarina* L. | 2 | Campanulaceae |
| *Cyphia* P. J. Bergius | 1 | Campanulaceae |
| *Lobelia* L. | 18 | Lobeliaceae |
| *Monopsis* Salisb. | 2 | Lobeliaceae |
| *Wahlenbergia* Schrad. ex Roth | 13 | Campanulaceae |
| f217. **Menyanthaceae** |  |  |
| *Nymphoides* Ség. | 2 | Menyanthaceae |
| f218. **Goodeniaceae** |  |  |
| *Scaevola* L. | 2 | Goodeniaceae |
| f219. **Asteraceae** |  |  |
| *Acanthospermum* Schrank | (2) | Compositae |
| *Achyrothalamus* O. Hoffm. | 1 | Compositae |
| *Acmella* Pers. | 2 (1) | Compositae |
| *Adenostemma* J. R. Forst. et G. Forst. | 3 | Compositae |
| *Ageratina* Spach | 1 | Compositae |
| *Ageratum* L. | (2) | Compositae |
| *Ambrosia* L. | (1) | Compositae |
| *Anisopappus* Hook. et Arn. | 2 | Compositae |
| *Anthemis* L. | 1 | Compositae |
| *Artemisia* L. | 1 | Compositae |
| *Artemisiopsis* S. Moore | 1 | Compositae |
| *Aspilia* Thouars | 4 | Compositae |
| *Athrixia* Ker-Gawl. | 1 | Compositae |
| *Athroisma* DC. | 5 | Compositae |
| *Berkheya* Ehrh. | 1 | Compositae |
| *Bidens* L. | 18 | Compositae |
| *Blainvillea* Cass. | 1 | Compositae |
| *Blepharispermum* Wight ex DC. | 5 | Compositae |
| *Blumea* DC. | 2 | Compositae |
| *Bothriocline* Oliv. ex Benth. | 10 | Compositae |
| *Brachylaena* R. Br. | 1 | Compositae |
| *Carduus* L. | 7 | Compositae |
| *Centaurea* L. | 1 (1) | Compositae |
| *Chrysanthellum* Rich. | 1 | Compositae |
| *Chrysanthemoides* Fabr. | 1 | Compositae |
| *Chrysanthemum* L. | (3) | Compositae |
| *Cichorium* L. | (1) | Compositae |
| *Cineraria* L. | 1 | Compositae |
| *Cirsium* Mill. | 2 | Compositae |
| *Conyza* Less. | 20 (1) | Compositae |
| *Coreopsis* L. | (1) | Compositae |
| *Cosmos* Cav. | 2 | Compositae |
| *Cotula* L. | 3 (1) | Compositae |
| *Crassocephalum* Moench | 6 | Compositae |
| *Crepis* L. | 3 (1) | Compositae |
| *Dahlia* Cav. | (2) | Compositae |
| *Delamerea* S. Moore | 1 | Compositae |
| *Dendrosenecio* (Hauman ex Hedberg) B. Nord. | 6 | Compositae |
| *Dianthoseris* Sch.-Bip. ex A. Rich. | 1 | Compositae |
| *Dichrocephala* L'Hér. ex DC. | 2 | Compositae |
| *Dicoma* Cass. | 1 | Compositae |
| *Distephanus* Cass. | 2 | Compositae |
| *Echinops* L. | 9 | Compositae |
| *Eclipta* L. | 1 | Compositae |
| *Elephantopus* L. | 1 | Compositae |
| *Emilia* (Cass.) Cass. | 12 | Compositae |
| *Enydra* Lour. | 1 | Compositae |
| *Erigeron* L. | 1 (1) | Compositae |
| *Erlangea* Sch.-Bip. | 3 | Compositae |
| *Erythrocephalum* Benth. et Hook. f. | 2 | Compositae |
| *Ethulia* L. f. | 6 | Compositae |
| *Euryops* (Cass.) Cass. | 4 | Compositae |
| *Felicia* Cass. | 4 | Compositae |
| *Flaveria* Juss. | 1 | Compositae |
| *Galinsoga* Ruiz et Pav. | (2) | Compositae |
| *Gamochaeta* Wedd. | 1 | Compositae |
| *Geigeria* Griess. | 2 | Compositae |
| *Gerbera* L. | 2 | Compositae |
| *Gnaphalium* L. | 2 | Compositae |
| *Grangea* Adans. | 1 | Compositae |
| *Grauanthus* Fayed | 1 | Compositae |
| *Guizotia* Cass. | 4 | Compositae |
| *Gutenbergia* Sch.-Bip. ex Walp. | 6 | Compositae |
| *Gynura* Cass. | 6 | Compositae |
| *Haplocarpha* Less. | 2 | Compositae |
| *Helianthus* L. | (1) | Compositae |
| *Helichrysum* Mill. | 24 (1) | Compositae |
| *Hirpicium* Cass. | 1 | Compositae |
| *Hypochaeris* L. | (1) | Compositae |
| *Inula* L. | 3 | Compositae |
| *Iphionopsis* A. Anderb. | 1 | Compositae |
| *Kleinia* Mill. | 18 | Compositae |
| *Lactuca* L. | 3 | Compositae |
| *Lagascea* Cav. | (1) | Compositae |
| *Laggera* Sch.Bip. ex Benth. et Hook. f. | 3 | Compositae |
| *Launaea* Cass. | 8 | Compositae |
| *Leucanthemum* Mill. | (1) | Compositae |
| *Litogyne* Harv. | 1 | Compositae |
| *Matricaria* L. | (1) | Compositae |
| *Melanthera* Rohr | 3 | Compositae |
| *Micractis* DC. | 1 | Compositae |
| *Microglossa* DC. | 3 | Compositae |
| *Mikania* Willd. | 2 | Compositae |
| *Mikaniopsis* Milne-Redh. | 2 | Compositae |
| *Nicolasia* S. Moore | 1 | Compositae |
| *Nidorella* Cass. | 3 | Compositae |
| *Osteospermum* L. | 2 | Compositae |
| *Parthenium* L. | (1) | Compositae |
| *Pegolettia* Cass. | 1 | Compositae |
| *Pluchea* Cass. | 4 | Compositae |
| *Porphyrostemma* Benth. ex Oliv. | 2 | Compositae |
| *Prenanthes* L. | 1 | Compositae |
| *Pseudoconyza* Cuatrec. | 1 | Compositae |
| *Pseudognaphalium* Kirp. | 2 | Compositae |
| *Psiadia* Jacq. | 2 | Compositae |
| *Pulicaria* Gaertn. | 1 | Compositae |
| *Reichardia* Roth | 1 | Compositae |
| *Schkuhria* Roth | 1 | Compositae |
| *Sclerocarpus* Jacq. | 1 | Compositae |
| *Scorzonera* L. | (1) | Compositae |
| *Senecio* L. | 27 (1) | Compositae |
| *Sigesbeckia* L. | 1 | Compositae |
| *Silybum* Vaill. ex Adans. | (1) | Compositae |
| *Solanecio* (Sch.-Bip.) Walp. | 8 | Compositae |
| *Sonchus* L. | 7 (1) | Compositae |
| *Sphaeranthus* L. | 12 | Compositae |
| *Spilanthes* Jacq. | 1 | Compositae |
| *Stoebe* L. | 1 | Compositae |
| *Stomatanthes* R. M. King et H. Rob. | 1 | Compositae |
| *Synedrella* Gaertn. | 1 | Compositae |
| *Tagetes* L. | (2) | Compositae |
| *Tanacetum* L. | (2) | Compositae |
| *Taraxacum* F. H. Wigg. | (1) | Compositae |
| *Tarchonanthus* L. | 1 | Compositae |
| *Thymophylla* Lag. | (1) | Compositae |
| *Tithonia* Desf. | (2) | Compositae |
| *Tolpis* Adans. | 1 | Compositae |
| *Tridax* L. | (1) | Compositae |
| *Triplocephalum* O. Hoffm. | 1 | Compositae |
| *Vernonia* Schreb. | 52 | Compositae |
| *Volutaria* Cass. | 1 (1) | Compositae |
| *Xanthium* L. | (1) | Compositae |
| *Zinnia* L. | (2) | Compositae |
| **Dipsacales** |  |  |
| f220. **Adoxaceae** |  |  |
| *Sambucus* L. | 1 | Caprifoliaceae |
| f221. **Caprifoliaceae** |  |  |
| *Cephalaria* Schrad. | 1 | Dipsacaceae |
| *Dipsacus* L. | 1 | Dipsacaceae |
| *Pterocephalus* Vaill. ex Adans. | 1 | Dipsacaceae |
| *Scabiosa* L. | 2 | Dipsacaceae |
| *Valeriana* L. | 3 | Valerianaceae |
| *Valerianella* Mill. | 1 | Valerianaceae |
| **Apiales** |  |  |
| f222. **Pittosporaceae** |  |  |
| *Pittosporum* Banks ex Gaertn. | 4 (1) | Pittosporaceae |
| f223. **Araliaceae** |  |  |
| *Cussonia* Thunb. ex Thunb. | 4 | Araliaceae |
| *Hydrocotyle* L. | 3 | Umbelliferae |
| *Polyscias* J. R. Forst. et G. Forst. | 3 | Araliaceae |
| *Schefflera* J. R. Forst. et G. Forst. | 3 | Araliaceae |
| f224. **Apiaceae** |  |  |
| *Agrocharis* Hochst. | 2 | Umbelliferae |
| *Alepidea* D. Delaroche | 1 | Umbelliferae |
| *Ammi* L. | (1) | Umbelliferae |
| *Anethum* L. | 1 | Umbelliferae |
| *Anthriscus* (Pers.) Hoffm. | 1 | Umbelliferae |
| *Apium* L. | 1 | Umbelliferae |
| *Berula* W. D. J. Koch | 1 | Umbelliferae |
| *Centella* L. | 1 | Umbelliferae |
| *Coriandrum* L. | 1 | Umbelliferae |
| *Cryptotaenia* DC. | 1 | Umbelliferae |
| *Diplolophium* Turcz. | 1 | Umbelliferae |
| *Erythroselinum* Chiov. | 1 | Umbelliferae |
| *Ferula* L. | 1 | Umbelliferae |
| *Foeniculum* Mill. | (1) | Umbelliferae |
| *Haplosciadium* Hochst. | 1 | Umbelliferae |
| *Heracleum* L. | 4 | Umbelliferae |
| *Heteromorpha* Cham. et Schltdl. | 1 | Umbelliferae |
| *Lefebvrea* A. Rich. | 2 | Umbelliferae |
| *Oenanthe* L. | 2 | Umbelliferae |
| *Oreoschimperella* Rauschert | 1 | Umbelliferae |
| *Peucedanum* L. | 9 | Umbelliferae |
| *Pimpinella* L. | 4 | Umbelliferae |
| *Pseudocarum* C. Norman | 1 | Umbelliferae |
| *Sanicula* L. | 1 | Umbelliferae |
| *Steganotaenia* Hochst. | 1 | Umbelliferae |
| *Torilis* Adans. | 1 | Umbelliferae |
| *Trachyspermum* Link | 1 (1) | Umbelliferae |
